# Biologic targets of prescription medications and risk of neurodegenerative disease in United States Medicare beneficiaries

**DOI:** 10.1371/journal.pone.0285011

**Published:** 2023-05-17

**Authors:** Yizhe Song, Brad A. Racette, Alejandra Camacho-Soto, Susan Searles Nielsen

**Affiliations:** 1 Department of Neurology, Washington University School of Medicine, St. Louis, Missouri, United States of America; 2 Department of Neurology, Barrow Neurological Institute, Phoenix, Arizona, United States of America; 3 Faculty of Health Sciences, School of Public Health, University of the Witwatersrand, Johannesburg, South Africa; 4 Department of Orthopedics, Washington University School of Medicine, St. Louis, Missouri, United States of America; Institut d’Investigacions Biomediques de Barcelona, SPAIN

## Abstract

**Objective:**

To identify prescription medications associated with a lower risk of three neurodegenerative diseases: Parkinson disease, Alzheimer disease, and amyotrophic lateral sclerosis.

**Methods:**

We conducted a population-based, case-control study of U.S. Medicare beneficiaries in 2009 (42,885 incident neurodegenerative disease cases, 334,387 randomly selected controls). Using medication data from 2006–2007, we categorized all filled medications according to their biological targets and mechanisms of action on those targets. We used multinomial logistic regression models, while accounting for demographics, indicators of smoking, and health care utilization, to estimate odds ratios (ORs) and 95% confidence intervals (CIs) for 141 target-action pairs and each neurodegenerative disease. For target-action pairs inversely associated with all three diseases, we attempted replication in a cohort study that included an active comparator group. We constructed the cohort by following controls forward for incident neurodegenerative disease from the beginning of 2010 until death or end of 2014, i.e., up to five years after the two-year exposure lag. We used Cox proportional hazards regression while accounting for the same covariates.

**Results:**

The most consistent inverse association across both studies and all three neurodegenerative diseases was for xanthine dehydrogenase/oxidase blockers, represented by the gout medication, allopurinol. Allopurinol was associated with a 13–34% lower risk for each neurodegenerative disease group in multinomial regression, and a mean reduction of 23% overall, as compared to individuals who did not use allopurinol. In the replication cohort we observed a significant 23% reduction for neurodegenerative disease in the fifth year of follow-up, when comparing allopurinol users to non-users, and more marked associations with an active comparator group. We observed parallel associations for a related target-action pair unique to carvedilol.

**Discussion/Conclusion:**

Xanthine dehydrogenase/oxidase blockade might reduce risk of neurodegenerative disease. However, further research will be necessary to confirm that the associations related to this pathway are causal or to examine whether this mechanism slows progression.

## Introduction

The global prevalence of neurodegenerative diseases, such as Parkinson disease (PD), Alzheimer disease (AD), and amyotrophic lateral sclerosis (ALS), is increasing due to an aging population and increasing life expectancy in most parts of the world [1–3]. Currently, treatments for neurodegenerative diseases are largely focused on symptom management making it urgent to identify disease-modifying therapies. Pharmaceutical treatments can emerge through the synthesis of new drugs or the repurposing of existing drugs. The latter approach is cost-effective as FDA-approved medications typically have well-established pharmacokinetic and safety profiles.

In this study, we used a large, population-based administrative claims dataset, specifically Part D pharmacy claims data from United States (U.S.) Medicare beneficiaries, to identify prescription drugs associated with a lower risk of PD, AD, and/or ALS. To maximize our ability to identify potentially protective drugs, we screened medications according to their biological targets and pharmacological actions on those targets, rather than as individual drugs. We hypothesized that the three neurodegenerative diseases share some common neurodegenerative mechanisms and that epidemiologic evidence of medications associated with a lower risk of multiple neurodegenerative diseases would represent high-priority candidates for disease-modifying clinical trials. This work suggested one biologic target as a priority for further research.

## Materials and methods

### Study overview

Based on U.S. Medicare beneficiaries ≥66 years of age with Part D pharmacy coverage (claims data), we constructed a large, population-based case-control study with incident AD, PD, and ALS cases in 2009, along with comparable beneficiaries without these conditions in the same year. Medicare is the national health insurance used by >98% of Americans age ≥65. Following identification of candidate neuroprotective medications from this case-control study, we attempted to replicate our strongest findings using the cohort design. Specifically, we constructed a large, Medicare-based follow-up study from 2010–2014 while applying both traditional and active comparator approaches. The study was approved by the Washington University Human Research Protection Office and the Centers for Medicare and Medicaid Services (CMS), which only released data after de-identification. Informed consent was not required in this records-based study, which was classified as not involving human subjects research; we obtained a waiver.

### Study eligibility criteria

All cases and controls met study criteria designed to ensure complete case ascertainment and a population-based sample of both cases and controls [4]. Briefly, both met all of the following criteria in 2009: 1) age ≥66 years, 11 months (age-eligible for Medicare ≥2 years) but ≤90, 2) Medicare Part A/B coverage and no Part C coverage, and 3) residence in the U.S. We determined the above from the Medicare summary file for 2009, which enumerates basic demographic and medical information for all beneficiaries. During this time period, approximately a quarter of beneficiaries otherwise eligible for our study had Part C coverage [4]. For the present study, we also required Medicare Part D (pharmacy) coverage and ≥1 prescription claim in 2006–2007. Part D is an optional Medicare program that covers prescription medications, which began in 2006. We required and restricted to Part D claims in these years because this approach ensured that all medications were first prescribed >1 year before PD/AD/ALS diagnosis (or control reference date) in 2009, in order to minimize detection of associations due to prodromal disease symptomatology. For the follow-up component, all of the same criteria applied. In addition, we required beneficiaries to have survived, without diagnosis of neurodegenerative disease, to January 1, 2010, the start of follow-up.

### Identification of PD, AD, and ALS cases

We identified PD, AD, and/or ALS on the basis of International Classification of Diseases, Ninth Revision, Clinical Modification (ICD-9-CM) diagnosis codes contained in comprehensive claims data in 2004–2009, specifically for PD (ICD-9-CM 332 or 332.0), AD (ICD-9-CM 331 or 331.0), and ALS (ICD-9-CM 335.2 or 335.20). In the case-control study, we restricted to incident cases in 2009, i.e., excluded prevalent cases in all years (2004–2009), by including only those PD/AD/ALS cases with a diagnosis code for the respective condition in 2009, but no prior years (2004–2008). We excluded from the PD case group 4.6% of potential cases with a diagnosis code for atypical parkinsonism (ICD-9-CM 333.0) or dementia with Lewy bodies (ICD-9-CM 331.82) [4]. We included all remaining incident PD cases who met study eligibility criteria. AD is the most common neurodegenerative disease, and we also included a 5% random sample of all incident AD cases who met study eligibility criteria. ALS is rare and we included all incident ALS cases who met study eligibility criteria. We applied the same case identification approach during follow-up in 2010–2014.

We identified 42,885 incident cases of PD/AD/ALS in 2009. In order to leverage independent findings across the three neurodegenerative conditions and identify the most promising neuroprotective drug candidates, we classified cases into one of four groups: PD only (N = 28,679), AD only (N = 8,332), ALS only (N = 1,341), or a “mixed” PD/AD/ALS case category (N = 4,533), containing a code for >1 of these conditions. We retained this mixed case group because beneficiaries who obtain a code for only one condition might differ from those in the mixed group in ways that are not restricted to certainty of (the single) diagnosis. In addition, this mixed group provided for a case group in which we hypothesized we could replicate associations that we observed across multiple individual case groups, particularly for PD and AD. During the five years of follow-up in 2010–2014 we identified 18,293 additional incident cases of neurodegenerative disease.

### Selection of controls

In the case-control study, controls were a random sample (N = 334,387) of all beneficiaries enumerated in the 2009 summary file who met the same criteria, but without any of the above ICD-9-CM diagnosis codes for PD, AD, ALS, other motor neuron disease (ICD-9-CM 335.21, 335.22, 335.23, 335.24, or 335.29), atypical parkinsonism, or dementia with Lewy bodies. Because our large sample size provided good statistical power, we opted to adjust for covariates rather than match controls to cases [5]. We used all controls for comparisons to each case group. In addition, a random sample of all controls who survived to January 1, 2010 were available for the follow-up study (N = 207,764). By definition these controls were free of neurodegenerative disease on that date.

### Assessment of medication use and their biological targets

We used Part D pharmacy claims data from 2006–2007 to identify all medications used by cases and controls in the study period, effectively applying >1 year of exposure lagging. We classified a beneficiary as having used a medication if there was ≥1 prescription event (fill) of the respective medication. We then identified the active ingredient(s) in each medication. In total, we identified use of 768 active ingredients. For each active ingredient, we next identified the biological targets, such as specified metabolic enzymes or receptors, and the pharmacological actions on each target, i.e., activator, blocker, or other. We identified these targets and actions through DrugBank [6]. Presented results are based on targets and actions in this database by February 2023, and we manually reviewed active ingredients in our dataset to ensure linkage when active ingredient names differed. The 768 active ingredients in our dataset corresponded to 723 targets from DrugBank, which we collapsed by similarity to 561 unique targets. We then simultaneously considered the pharmacological action on each target, yielding 723 biological target-action pairs. Of these, 141 target-action pairs had >10 beneficiaries in each case group and in the control group (S1 Table in S1 File), and we focused on these most commonly observed target-action pairs to meet CMS reporting requirements.

### Assessment of covariates

We obtained each beneficiary’s birth date (age), sex, and race/ethnicity from the 2009 Medicare summary file. We determined indicators of overall use of medical care from 2008, so that post-diagnosis care would not be included. Use of care is a strong potential confounder of the association between various medical conditions and PD [7], and therefore also is a potential confounder in a pharmacoepidemiologic study. As direct indicators of use of care we obtained the number of outpatient/physician (carrier) visits and number of days hospitalized as an inpatient including in a skilled nursing facility from the summary file from 2008. From these same summary files in 2007–2008 we used a count of all medical conditions tracked by the Chronic Conditions Data Warehouse (CCW) other than AD and sex-specific conditions. These included acute myocardial infarction, rheumatoid/osteoarthritis, atrial fibrillation, cataract, cancer of the colon, cancer of the lung, chronic kidney disease, chronic obstructive pulmonary disease (COPD), congestive heart failure, depression, diabetes, fracture of the hip/pelvis, glaucoma, ischemic heart disease, osteoporosis, and stroke/transient ischemic attack. These 16 conditions largely overlap with those used to calculate the Charlson Comorbidity Index, and as such, the number of these conditions can serve as an indirect indicator of use of medical care. In addition to this sum of medical conditions as an a priori covariate, we retained a single dichotomous variable for each of these 16 conditions as additional covariates available for post-hoc sensitivity analyses (selected according to potential indications for the respective prescribed medications). We also combined the COPD and lung cancer variables as an indicator of smoking, given that most individuals with COPD or lung cancer are current or previous tobacco smokers. We had no missing covariate data, with the exception of race/ethnicity for a small percentage of beneficiaries who we combined with those of an “other” race/ethnicity.

### Statistical analysis

We used R version 4.2.1 to prepare figures and otherwise conducted all analyses using Stata/MP version 14.2 or 17.0 [8]. In our case-control study, we used multinomial (polytomous) logistic regression to estimate adjusted odds ratios (ORs) and 95% confidence intervals (CIs) between each target-action pair and each neurodegenerative disease group relative to all controls. We generated one model per target-action pair, with the outcome in five categories (PD only, AD only, ALS only, mixed PD/AD/ALS cases, or none of these outcomes [controls]). In the follow-study, we used Cox proportional hazards regression in our primary analysis and competing-risks survival analysis in sensitivity analysis, as detailed below.

In all analyses, we adjusted *a priori* for age, as a continuous variable, using Harrell’s method [9], to allow for flexible adjustment, recognizing that the shape of the strong association between age and each of the three neurodegenerative conditions differs. We also adjusted for sex and race/ethnicity (White, Black, Hispanic, and Asian/Pacific Islander [API]/other/unknown), use of medical care, and number of CCW conditions (as detailed above, each as a continuous variable), and a dichotomous indicator of smoking (COPD or lung cancer). In addition, we confirmed all reported associations in sensitivity analyses by simultaneously adjusting for dichotomous variables for selected CCW medical conditions and medications that could potentially confound the association of interest.

Because we aimed to identify neuroprotective medications, we primarily report inverse associations from our discovery step, i.e., case-control study. To avoid missing associations due to insufficient statistical power, we used two-sided α = 0.05 to determine significance, rather than correcting for multiple comparisons. Instead, we considered both the magnitude and consistency of the associations across the four case groups. As a measure of both magnitude and consistency, we calculated the mean OR across these four case groups for each biological target-action pair. To explore the consistency of the potential inverse associations for a target-action pair further, we examined associations for the individual medications in the respective target-action pair, as well as by sex in sex-specific models (two models per target-action pair). In the cohort analyses, we focused on selected candidate medications and pre-hypothesized the direction of effect, but retained our more conservative two-sided α = 0.05.

We attempted to replicate the strongest inverse associations in a cohort study that included an active comparator group [10], i.e., individuals who use different medications with the same indication as the medication of interest. This approach is used to address confounding by indication and ensure greater comparability of the groups. This approach can also be combined with the new user design [10]. However, we did not simultaneously apply the new user design because of the known diagnostic delay in ALS [11] and the long prodromal period for AD and PD [12], which together could imply that the observed exposures could not have occurred before disease onset. Rather, similar to the initial study, we applied a two-year exposure lag to exclude medications first used between disease onset and diagnosis. In this cohort we followed the sample of controls who survived to January 1, 2010 forward for incident neurodegenerative disease until death or end of 2014, i.e., up to five years. We also censored individuals if and when they developed atypical parkinsonism, developed dementia with Lewy bodies, or enrolled in Part C coverage. Total time at risk and under observation was 781,486 years in this study. We used Cox proportional hazards regression, while accounting for the same covariates as of baseline, both with and without relevant CCW medical conditions and other medications. We used age as the time scale [13] to account for age and then included all other covariates as independent variables in the model. We estimated hazards ratios (HRs) and 95% CIs from the Cox model. We checked the proportional hazards assumption using Schoenfeld residuals [14]. In sensitivity analyses we excluded PD/AD/ALS in progressively more years of follow-up to further minimize potential effects of reverse causation, given that only two years of exposure lagging was possible because Part D coverage first became available to Medicare beneficiaries in 2006. In additional sensitivity analyses we conducted competing-risks survival regression based on Fine and Gray’s proportional subhazards model [15]. In these analyses we treated the start of Part C coverage and death as competing risks.

Because PD, AD, and ALS are all rare, and because we did not use incidence density sampling of controls, we interpreted ORs from our case-control study as relative risks, rather than as ORs or incidence rate ratios [16]. The latter are most comparable to HRs, but for simplicity we interpreted HRs from our cohort study as approximations of relative risk over the five years of follow-up when the proportional hazards assumption was met.

## Results

### Participant characteristics

In each case group and in controls, a majority (>80%) of beneficiaries were non-Hispanic white, and >50% were female (Table 1). PD and ALS were each more common in men than women, and in non-Hispanic Whites than each of the other racial/ethnic groups. In contrast, AD was more common in women than in men, and more common in Black and Hispanic beneficiaries than in White or especially Asian/Pacific Islander beneficiaries, who had the lowest AD risk. Risk of PD and AD increased with age, whereas risk of ALS peaked in the 70s. When accounting for these demographic differences, history of smoking (COPD or lung cancer) was less common in PD cases as compared to controls. In the 1–2 years prior to diagnosis with a neurodegenerative condition, use of medical care in cases was generally greater than among controls.

**Table 1:** Characteristics of beneficiaries with and without incident PD, AD, and/or ALS, U.S. Medicare 2009.

	PD only N = 28,679 %	OR (95% CI)^a^	AD only N = 8,332 %	OR (95% CI)^a^	ALS only N = 1,341 %	OR (95% CI)^a^	Mixed PD/AD/ ALS N = 4,533 %	OR (95% CI)^a^	Controls N = 334,387 %
**Age, years**									
66–69	8.1	1.0 (Reference)	3.7	1.0 (Reference)	12.5	1.0 (Reference)	4.8	1.0 (Reference)	13.6
70–74	22.6	1.20 (1.14, 1.26)	12.5	1.47 (1.29, 1.67)	31.8	1.13 (0.95, 1.36)	14.2	1.29 (1.11, 1.51)	29.9
75–79	25.0	1.58 (1.51, 1.66)	21.0	2.97 (2.63, 3.35)	23.2	1.02 (0.84, 1.23)	23.2	2.51 (2.17, 2.91)	23.7
80–90	44.2	1.90 (1.81, 1.99)	62.8	5.91 (5.26, 6.63)	32.5	1.02 (0.85, 1.23)	57.8	4.18 (3.63, 4.81)	32.9
Mean (SD)	78.4 (6.1)	—	81.0 (5.9)	—	76.6 (6.1)	—	80.4 (5.9)	—	76.6 (6.2)
**Sex**									
Male	43.1	1.0 (Reference)	28.8	1.0 (Reference)	45.4	1.0 (Reference)	39.1	1.0 (Reference)	35.6
Female	56.9	0.65 (0.64, 0.67)	71.2	1.14 (1.09, 1.20)	54.6	0.64 (0.58, 0.72)	60.9	0.72 (0.68, 0.76)	64.4
**Race/ethnicity**									
White	86.7	1.0 (Reference)	80.6	1.0 (Reference)	88.1	1.0 (Reference)	81.3	1.0 (Reference)	84.1
Black	5.8	0.74 (0.71, 0.78)	10.5	1.43 (1.33, 1.54)	6.6	0.84 (0.68, 1.05)	8.7	1.18 (1.06, 1.31)	7.7
Hispanic	2.8	0.91 (0.84, 0.98)	4.3	1.47 (1.31, 1.64)	1.3	0.47 (0.30, 0.75)	4.8	1.61 (1.40, 1.85)	2.7
API/other/unknown	4.7	0.85 (0.80, 0.90)	4.6	0.91 (0.82, 1.01)	3.9	0.69 (0.52, 0.91)	5.2	1.00 (0.88, 1.15)	5.5
**Use of medical care** ^b^									
Annual number of physician/outpatient visits, mean (SD)	16.2 (12.9)	1.006 (1.006, 1.007)	13.4 (11.7)	0.995 (0.994, 0.996)	17.1 (13.8)	1.012 (1.010, 1.014)	14.5 (12.3)	0.998 (0.997, 1.000)	12.5 (10.9)
Annual number of days in a hospital or SNF, mean (SD)	4.9 (17.6)	1.007 (1.007, 1.008)	6.3 (20.1)	1.009 (1.008, 1.010)	3.4 (12.8)	1.005 (1.002, 1.08)	6.9 (21.3)	1.009 (1.008, 1.011)	1.8 (9.9)
**Number of selected medical conditions** ^b^ ^,c^	3.5 (2.1)	1.16 (1.15, 1.17)	3.5 (2.1)	1.14 (1.13, 1.16)	3.0 (2.0)	1.02 (0.99, 1.06)	3.6 (2.2)	1.20 (1.18, 1.22)	2.7 (1.9)
**COPD or lung cancer** ^b^	20.5	0.86 (0.83, 0.89)	20.7	0.99 (0.93, 1.06)	20.4	1.10 (0.95, 1.29)	20.7	0.85 (0.78, 0.92)	15.3

^a^ Adjusted for all variables shown. ORs for use of care/conditions are per visit, day, or condition. ORs for COPD/lung cancer are relative to beneficiaries without COPD and lung cancer.

^b^ Prior to diagnosis, specifically in 2008 (visits or days) or 2007–2008 (medical conditions, including COPD and lung cancer).

^c^ Of all (16) conditions available from the 2007–2008 Medicare beneficiary annual summary files other than AD/dementia and sex-specific cancers: Acute myocardial infarction, rheumatoid/osteoarthritis, atrial fibrillation, cataract, cancer of the colon, cancer of the lung, chronic kidney disease, chronic obstructive pulmonary disease (COPD), congestive heart failure, depression, diabetes, fracture of the hip/pelvis, glaucoma, ischemic heart disease, osteoporosis, and stroke/transient ischemic attack.

^c^ Of all (16) conditions available from the 2007–2008 Medicare beneficiary annual summary files other than AD/dementia and sex-specific cancers: Acute myocardial infarction, rheumatoid/osteoarthritis, atrial fibrillation, cataract, cancer of the colon, cancer of the lung, chronic kidney disease, chronic obstructive pulmonary disease (COPD), congestive heart failure, depression, diabetes, fracture of the hip/pelvis, glaucoma, ischemic heart disease, osteoporosis, and stroke/transient ischemic attack.

### Neurodegenerative disease associations in the case-control (discovery) study

**Fig 1:**
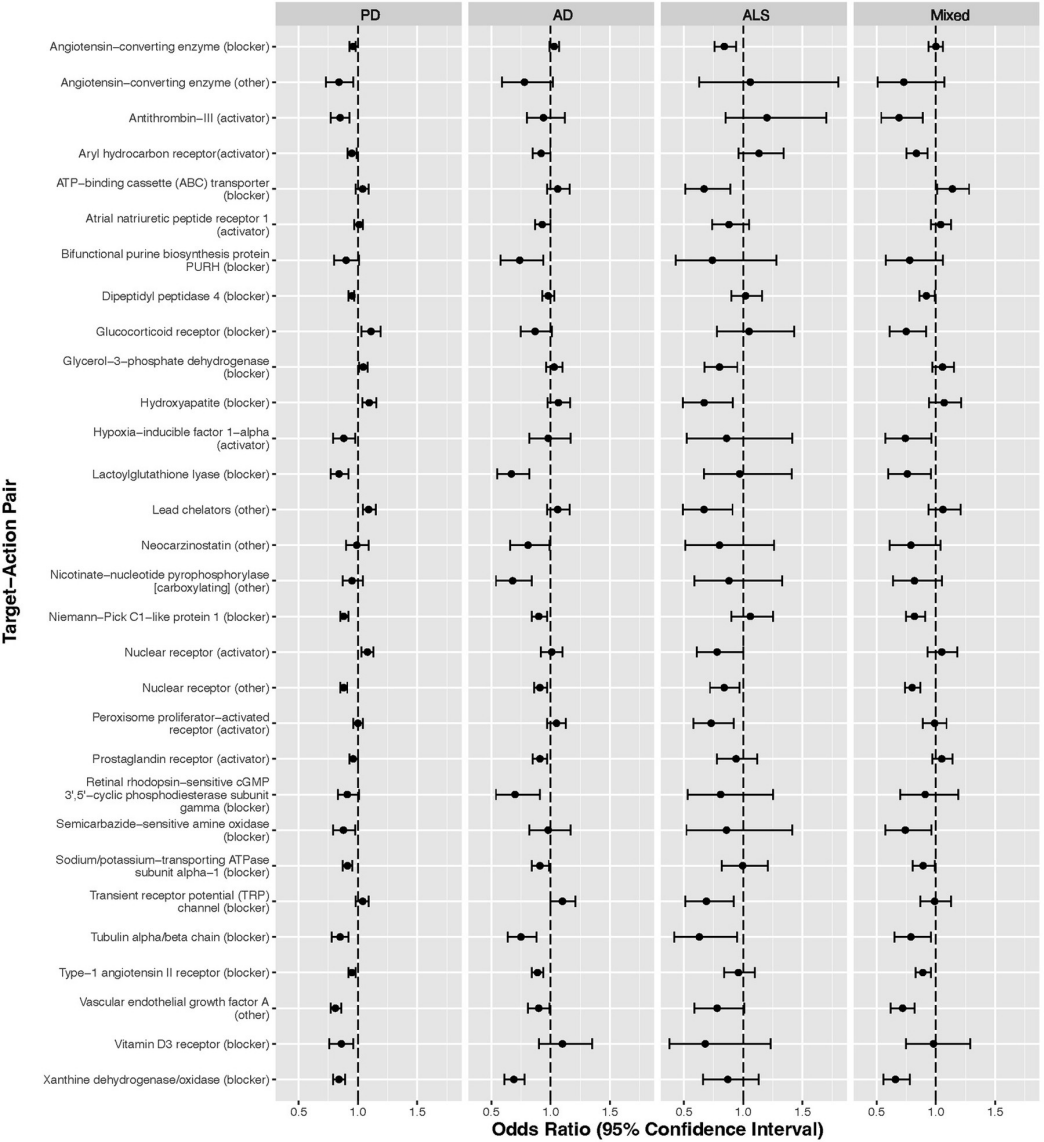
Medication target-action pairs significantly inversely associated with at least one neurodegenerative condition, U.S. Medicare 2009. **Legend:** Thirty target-action pairs were significantly associated with lower risk of ≥1 neurodegenerative disease. Four or these target-action pairs had a mean odds ratio ≤0.80 (≥20% average reduction in risk) across all four case groups (Table 2) and the remainder had a mean odds ratio <1.00 (S2 Table in S1 File).

Thirty target-action pairs were significantly associated with lower risk of ≥1 neurodegenerative disease (Table 2 and Fig 1 and S2 Table in S1 File). Four of these target-action pairs had a mean OR ≤0.80 (≥20% average reduction in risk) across all four case groups (Table 2). All four neurodegenerative case groups were significantly inversely associated with one target-action pair, tubulin alpha/beta chain blockers (represented by six medications, including the anti-gout medication colchicine, target-action pair mean OR = 0.76). However, this target-action pair association appeared stronger in men (mean OR = 0.67) than women (mean OR = 0.90), and was inconsistent across the individual medications within this target-action pair. Specifically, there was no evidence of lower risk of any of the neurodegenerative conditions in relation to any of the tubulin alpha/beta chain blockers other than colchicine. Use of three of these medications was too infrequent to consider individually, while the OR point estimates for the remaining tubulin alpha/beta chain blockers were universally at or above null for all four neurodegenerative conditions (ORs ranged from 0.99 to 1.40 with a mean of 1.20 for griseofulvin, and ORs ranged from 0.97 to 2.06 with a mean of 1.55 for mebendazole).

**Table 2:** Medication target-action pairs significantly inversely associated with neurodegenerative condition(s) and ≥20% average reduction in relative risk, U.S. Medicare 2009.

		Adjusted OR (95% CI)^a^				
Target (action)	Medication(s)	PD only N = 28,679	AD only N = 8,332	ALS only N = 1,341	Mixed PD/AD/ALS N = 4,533	Mean OR
Tubulin alpha/beta chain (blocker)	Albendazole, colchicine, griseofulvin, mebendazole, paclitaxel, podofilox	**0.85 (0.78, 0.92)**	**0.75 (0.64, 0.88)**	**0.63 (0.42, 0.95)**	**0.79 (0.65, 0.96)**	0.76
Xanthine dehydrogenase/oxidase (blocker)	Allopurinol^b^	**0.84 (0.79, 0.89)**	**0.69 (0.61, 0.78)**	0.87 (0.66, 1.13)	**0.66 (0.56, 0.78)**	0.77
Bifunctional purine biosynthesis protein PURH (blocker)	Methotrexate	0.90 (0.80, 1.01)	**0.74 (0.58, 0.94)**	0.74 (0.43, 1.28)	0.78 (0.58, 1.06)	0.79
Vascular endothelial growth factor A (other)^b^	Carvedilol^b^	**0.81 (0.77, 0.86)**	**0.90 (0.81, 0.99)**	0.78 (0.59, 1.01)	**0.72 (0.62, 0.82)**	0.80

^a^ OR comparing beneficiaries who did vs. did not use any of the listed medications prior to diagnosis/reference, adjusted for age (continuous, using Harrell’s method), sex, race/ethnicity (4 categories: White, Black, Hispanic, and API/other/unknown), use of medical care (total number of physician/outpatient visits, total number of days in a hospital/skilled nursing facility, and total number of selected medical conditions, each prior to case diagnosis or control reference as continuous variables), and indicator of smoking (lung cancer or COPD).

^b^ Carvedilol also blocks effects of xanthine dehydrogenase/oxidase, but this is an off-target effect, so is not classified as such here.

Abbreviations: AD = Alzheimer disease; ALS = amyotrophic lateral sclerosis; API = Asian/Pacific Islander; COPD = chronic obstructive pulmonary disease; CI = confidence interval; OR = odds ratio; PD = Parkinson disease.

The three other target-action pairs with inverse associations suggested across all four case groups and with a mean OR ≤0.80 were represented by one medication each: xanthine dehydrogenase/oxidase blockers (the anti-gout medication allopurinol, mean OR = 0.77), bifunctional purine biosynthesis protein PURH blockers (methotrexate, typically used to treat psoriasis, rheumatoid arthritis, and cancer, mean OR = 0.79), and targets of vascular endothelial growth factor A (the beta blocker carvedilol, used for hypertension and heart failure, mean OR = 0.80) (Table 2). Results were relatively similar in men and women for both allopurinol (mean OR = 0.75 and 0.79, respectively) and carvedilol (mean OR = 0.79 and 0.81, respectively). OR point estimates for both men and women for all four case groups were <1.0 for both allopurinol and carvedilol, although some CIs were quite wide. Methotrexate use was uncommon in men, but for all case groups with >10 male cases, ORs in men and women were similar (PD ORs 0.83–0.88; AD ORs 0.75–0.76).

The remaining 26 target-action pairs with at least one significant inverse association had more modest mean ORs across the four case groups (Fig 1 and S2 Table in S1 File). Among these target-action pairs were hypoxia-inducible factor 1-alpha activators due to the inverse associations for carvedilol, as well as for hydralazine (captured alone in another target-action pair here). In addition, one target-action pair demonstrated significant inverse associations for all four neurodegenerative disease groups, medications with “other” effects on nuclear receptor (represented by ten medications including atorvastatin, flutamide, ketoconazole, spironolactone, warfarin, and selected chemotherapeutic agents, mean OR = 0.86). We confirmed this association in both men (mean OR = 0.83) and women (mean OR = 0.87). We observed less consistent results across disease groups for most of the remaining target-action pairs, including the lactoylglutathione lyase blocking medication, indomethacin, and targets of angiotensin-converting enzyme (chloroquine and hydroxychloroquine), which were not associated with ALS (Fig 1 and S2 Table in S1 File). Frequently, results were consistently inverse for both PD and AD but not ALS, or were only inverse for ALS. The latter associations were most commonly for target-action pairs that included medications used to treat type 2 diabetes or hypercholesterolemia. Among the target-action pairs with a mean OR≥1.0 (S3 Table in S1 File), none were in agreement or contrast with the associations noted above for xanthine oxidase/dehydrogenase (use of activators was too rare to examine) or bifunctional purine biosynthesis protein PURH (no other approved medications are known to otherwise target this pathway). In contrast to the targets mentioned above and an off-target blockade of effects from xanthine oxidase/dehydrogenase, target-action pairs for carvedilol (adrenergic receptor and potassium channel blocking) were not inversely associated with PD, AD, or ALS.

Insofar as we could apply further adjustments, the associations of greatest interest were not altered markedly by adjustment for the appropriate available CCW conditions or other medications with a strong confounding potential. When we adjusted allopurinol associations for colchicine, indomethacin, and rheumatoid/osteoarthritis (CCW variable closest to gout), ORs for each of the four neurodegenerative groups and allopurinol were attenuated by 5–9%. When we adjusted carvedilol associations for other beta blockers and cardiovascular disease, or adjusted methotrexate associations for rheumatoid/osteoarthritis, chloroquine, and hydroxychloroquine, ORs were altered up or down by ≤4%.

### Neurodegenerative disease associations in the cohort (replication) study

Given the robustness of the associations for allopurinol, carvedilol, and methotrexate, we selected all three for potential replication in our follow-up study. For allopurinol, we selected colchicine and indomethacin as active comparator medications. These medications were the ones most commonly prescribed gout in the Medicare formulary at the time we conducted our study. For carvedilol, we selected all other systemic (non-ophthalmic) beta blockers observed in our data: acebutolol, atenolol, betaxolol, bisoprolol, labetalol, metoprolol, nadolol, penbutolol, pindolol, propranolol, and sotalol. We focused on beta blockers rather than all possible medications that can be used for hypertension/cardiovascular disease in order to ensure that the active comparator group was as similar as possible to the carvedilol group. Finally, for methotrexate our active comparator medications were the following medications observed in our data that are used to treat rheumatoid arthritis: hydroxychloroquine, chloroquine, sulfasalazine, leflunomide, etanercept, and infliximab.

When we examined the associations between the three candidate medications and any PD/AD/ALS in the replication cohort study, our primary findings, i.e., for allopurinol and carvedilol, were largely confirmed. We again observed clear inverse associations for allopurinol (Table 3). HR point estimates for allopurinol were consistently inverse, even when restricted to users of allopurinol who did not use other gout medications. In addition, HR point estimates grew further below null when using an active comparator group or with greater exclusion of PD/AD/ALS diagnosed in the first years of follow-up. After excluding diagnoses in the first four years of follow-up, the HR most comparable to our initial analysis (HR = 0.77, 95% CI 0.62, 0.95) was essentially same as the mean OR across all PD/AD/ALS case groups from the initial analysis (OR = 0.77) (Tables 2 and 3). The same was true for carvedilol (HR = 0.81, 95% CI 0.67, 0.99 vs. mean OR = 0.80) (Table 2; S4 Table in S1 File). In contrast, we were unable to confirm an association for methotrexate (all HRs ≥0.87 and with wide CIs). Competing-risks survival regression did not materially alter conclusions for any of the three medications. Relative to the non-exposed comparator group, use of colchicine/indomethacin without allopurinol was not associated with neurodegenerative disease (HR = 1.02, 95% CI 0.92, 1.12) and risk of neurodegenerative disease remained strongly inversely associated with allopurinol use both with (HR = 0.80, 95% 0.69, 0.91) and without (HR = 0.83, 95% CI 0.75, 0.92) the use of other gout medications. Beta blockers other than carvedilol were not associated with neurodegenerative disease either alone (HR = 0.99, 95% CI 95, 1.02) or in combination with carvedilol (HR = 0.96, 95% CI 0.85, 1.08), whereas carvedilol alone was associated with markedly lower risk (HR = 0.82, 95% CI 0.75, 0.90) as compared to the non-exposed comparator group.

**Table 3:** Allopurinol, other gout medications, and risk of neurodegenerative disease, U.S. Medicare 2010–2014.

PD/AD/ALS in specified years of follow-up		Total	Incident PD/AD/ALS	Allopurinol category		Any allopurinol	
				Non-exposed comparator HR (95% CI)^a^	Active comparator HR (95% CI)^a^	Basic model HR (95% CI)^a^	Full model HR (95% CI)^a^^,^^b^
**Years 1–5 (all years)**		**N = 207,764** **n (%)**	**N = 18,293** **n (%)**				
	No gout medications	195,264 (94.0)	17,208 (94.1)	1.0 (Reference)	**—**	1.0 (Reference)	1.0 (Reference)
	Colchicine/indomethacin only	4,586 (2.2)	437 (2.4)	1.05 (0.96, 1.16)	1.0 (Reference)		
	Allopurinol and colchicine/indomethacin	2,610 (1.3)	213 (1.2)	0.88 (0.77, 1.004)	**0.83 (0.71, 0.98)**	**0.86 (0.80, 0.93)**	**0.86 (0.79, 0.93)**
	Allopurinol only	5,304 (2.6)	435 (2.4)	**0.86 (0.78, 0.95)**	**0.82 (0.71, 0.93)**		
**Years 2–5**		**N = 203,470** ^c^ **n (%)**	**N = 13,999** **n (%)**				
	No gout medications	191,229 (93.4)	13,173 (94.1)	1.0 (Reference)	**—**	1.0 (Reference)	1.0 (Reference)
	Colchicine/indomethacin only	4,479 (2.2)	330 (2.4)	1.05 (0.94, 1.17)	1.0 (Reference)		
	Allopurinol and colchicine/indomethacin	2,550 (1.3)	153 (1.1)	**0.85 (0.72, 0.99)**	**0.80 (0.66, 0.98)**	**0.88 (0.80, 0.96)**	**0.88 (0.80, 0.96)**
	Allopurinol only	5,212 (2.6)	343 (2.5)	**0.89 (0.80, 0.99)**	**0.85 (0.73, 0.99)**		
**Years 3–5**		**N = 199,496** ^c^ **n (%)**	**N = 10,025** **n (%)**				
	No gout medications	187,495 (94.0)	9,439 (94.2)	1.0 (Reference)	**—**	1.0 (Reference)	1.0 (Reference)
	Colchicine/indomethacin only	4,391 (2.2)	242 (2.4)	1.09 (0.96, 1.23)	1.0 (Reference)		
	Allopurinol and colchicine/indomethacin	2,504 (1.3)	107 (1.1)	0.84 (0.69, 1.02)	**0.77 (0.62, 0.97)**	**0.86 (0.77, 0.95)**	**0.85 (0.76, 0.95)**
	Allopurinol only	5,106 (2.6)	237 (2.4)	**0.87 (0.76, 0.99)**	**0.80 (0.67, 0.96)**		
**Years 4–5**		**N = 195,717** ^c^ **n (%)**	**N = 6,246** **n (%)**				
	No gout medications	183,941 (94.0)	5,885 (94.2)	1.0 (Reference)	**—**	1.0 (Reference)	1.0 (Reference)
	Colchicine/indomethacin only	4,298 (2.2)	149 (2.4)	1.09 (0.92, 1.28)	1.0 (Reference)		
	Allopurinol and colchicine/indomethacin	2,470 (1.3)	73 (1.2)	0.94 (0.74, 1.18)	0.86 (0.65, 1.14)	**0.86 (0.75, 0.98)**	**0.85 (0.73, 0.98)**
	Allopurinol only	5,008 (2.6)	139 (2.2)	**0.82 (0.70, 0.98)**	**0.76 (0.60, 0.96)**		
**Year 5**		**N = 192,514** ^c^ **n (%)**	**N = 3,043** **n (%)**				
	No gout medications	180,930 (94.0)	2,874 (94.5)	1.0 (Reference)	**—**	1.0 (Reference)	1.0 (Reference)
	Colchicine/indomethacin only	4,226 (2.2)	77 (2.5)	1.16 (0.92, 1.45)	1.0 (Reference)		
	Allopurinol and colchicine/indomethacin	2,423 (1.3)	26 (0.9)	0.70 (0.47, 1.03)	**0.60 (0.39, 0.94)**	**0.77 (0.62, 0.95)**	**0.77 (0.62, 0.96)**
	Allopurinol only	4,935 (2.6)	66 (2.2)	0.81 (0.63, 1.03)	**0.70 (0.50, 0.97)**		

^a^ With age as the time scale to account for age, and adjusted sex, race/ethnicity (4 categories), use of medical care (number of physician/outpatient visits, number of days in a hospital/skilled nursing facility, and number of selected medical conditions, each prior to case diagnosis or control reference as continuous variables), and indicator of smoking (lung cancer or chronic obstructive pulmonary disease).

^b^ Also adjusted for rheumatoid arthritis/osteoarthritis, colchicine, and indomethacin.

^c^ Excludes incident PD/AD/ALS in the prior year(s) of follow-up.

Abbreviations: AD = Alzheimer disease; ALS = amyotrophic lateral sclerosis; CI = confidence interval; HR = hazard ratio; PD = Parkinson disease.

## Discussion

In this large, population-based study, we took a unique approach to investigate the association between various medication categories, as defined by biological target and pharmacological action on that target, in relation to the three most common neurodegenerative diseases: AD, PD, and ALS. These findings suggest a possible new direction for repurposing or developing medications for neuroprotection. Medications that block xanthine dehydrogenase/oxidase (allopurinol) or inhibit the enzyme’s effects (carvedilol) [17] were inversely associated with all three diseases, even though the two medications in this pathway have different indications. Carvedilol is used for hypertension and heart disease, while allopurinol is used to lower uric acid levels, mainly for gout. Residual confounding by smoking could create an inverse association between carvedilol and PD, but prior analyses demonstrate that the inverse carvedilol-PD association is unlikely due to confounding by either smoking or indication [18,19]. In our present work we also demonstrated that other medications commonly used for gout, colchicine and indomethacin, are unlikely to cause the inverse associations for allopurinol. First, we demonstrated that the inverse association between allopurinol and neurodegenerative disease was not confounded by either of these medications. Second, of several medications that block tubulin alpha/beta chain, colchicine was the only one inversely associated with neurodegenerative disease, further confirming that the association for colchicine is not causal, but confounded by indication or allopurinol. Confounding by indication as a potential cause of the allopurinol association is more difficult to rule out, as gout is inversely associated with all three neurodegenerative diseases [20–28], and allopurinol is primarily used for gout. Thus, although three prior studies reported inverse associations between allopurinol and PD, similar in magnitude to ours [29–31], confounding by indication remains universally possible. However, a Mendelian randomization study found that serum uric acid and PD are not associated [32]. In addition, randomized trials of inosine to increase serum uric acid failed to slow progression of PD [33,34], which could suggest that previously observed inverse gout-PD associations were due to allopurinol rather than uric acid. Both arguments against confounding by indication also apply to ALS [32,35]. With regard to AD, Mendelian randomization studies conflict as to whether uric acid and AD are associated [32,36], but we note that the inverse allopurinol association was particularly strong for AD in our study. Finally, in our cohort analyses we confirmed the association between allopurinol and neurodegenerative disease in an active comparator design. Taken together, these findings provide potential new insights to long-established observations and suggest possible new directions for repurposing or developing medications for neuroprotection.

The other most consistent finding across the three neurodegenerative diseases in our case-control analysis was a potentially protective effect of bifunctional purine biosynthesis protein PURH blockers. This target-action pair is represented by a single medication (methotrexate), which is indicated for different conditions (rheumatoid arthritis, psoriasis, and cancer) typically than either allopurinol or carvedilol. We previously found no evidence of confounding by indication for an inverse methotrexate-PD association in our population [37]. Another study found a stronger inverse methotrexate-PD association [30], but did not observe the association when restricting to rheumatoid arthritis patients [38]. Still, another study restricted to rheumatoid arthritis patients did find an inverse methotrexate-dementia association very similar to our association for AD [39]. Thus, findings for methotrexate are mixed both in the literature and in our own work, given the lack of association with neurodegenerative disease in our cohort analysis. Nonetheless, methotrexate, as a PURH blocker, remains of possible interest in light of our findings for the xanthine dehydrogenase/oxidase pathway, since PURH catalyzes the last two steps in the biosynthetic pathway that serves as the primary source of purine nucleotides [40]. As part of this process, PURH generates inosine monophosphate, a component of hypoxanthine. Xanthine dehydrogenase, and a closely related derivative enzyme, xanthine oxidase, are involved in purine metabolism. Xanthine dehydrogenase catalyzes the transformation of xanthine to uric acid. Xanthine oxidase catalyzes the same reaction, as well as the transformation of hypoxanthine to the purine xanthine, creating reactive oxygen species in both processes [41]. Accordingly, allopurinol, carvedilol, and methotrexate potentially reduce reactive oxygen species (by either limiting reactions catalyzed by xanthine oxidase or the hypoxanthine precursor to these reactions) or their effects [17].

Ultimately, further experimental and epidemiologic studies will be required to understand whether our findings are causal and related. Carvedilol protects not only against xanthine oxidase mediated oxidative stress [17], but also crosses the blood-brain barrier and protects against lipid peroxidation in brain homogenates due to its carbazole moiety [17,42]. Carvedilol possibly also protects against oxidative stress via an effect on hypoxia inducible factor 1-alpha [43]. Similarly, higher serum uric acid levels might lower risk and/or progression of neurodegenerative diseases by protecting neurons through anti-oxidant mechanisms [26–28,44]. Thus, it is possible that our findings are indicative of a critical role for oxidative stress in neurodegenerative disease rather than the importance of allopurinol and the xanthine oxidase/dehydrogenase pathway.

Key strengths of our analyses were the restriction to incident cases and application of exposure lagging through our exclusion of medications used in the years immediately prior to diagnosis with a neurodegenerative disease. Despite these strengths and our intriguing findings, we must note some potential limitations to this administrative data study. Our findings are based on Medicare beneficiaries >65 years old and may not be generalizable to those with younger onset of neurodegenerative disease. In addition, because the definition of neurodegenerative diseases was based on administrative diagnosis codes, our case ascertainment method may have resulted in misclassification of some beneficiaries due to misdiagnosis or lack of diagnosis. Nonetheless, the demographic characteristics of our cases relative to controls were similar to that seen in other published studies [45–48], providing some reassurance that our case groups were sufficiently accurate to provide meaningful results. We also could not fully investigate the time course of disease and long-term use of medications as they relate to disease onset because we were limited to prescription claims data from the most recent years prior to neurodegenerative disease diagnosis. As such, we may have misclassified some beneficiaries as not taking a medication if they stopped using it before our study period. The above measurement error in both our outcome and exposure variables would likely tend to bias ORs toward null. Thus, we might have missed some true associations, and the ones we report are likely stronger than observed. With that caveat, we recognize that the potential neuroprotective effects we observed were modest. We also might have missed some true associations due to combining medications based on biological target-action pairs, rather than chemical structure, which also could have biological effects and be relevant to neurodegenerative disease risk. Finally, the medication associations we studied relate to disease risk, rather than progression, yet medications that impact disease risk may not necessarily impact disease progression.

In conclusion, by classifying medications according to their specific action on their biological targets, we identified a target-action pair associated with a lower risk of developing PD, AD, and ALS. Blockade of xanthine dehydrogenase/oxidase or its effects, regardless of indication, was associated with a modestly reduced risk of all three neurodegenerative diseases. This mechanism, and possibly also related mechanisms as suggested by our secondary findings for bifunctional purine biosynthesis protein PURH, may warrant further study as a potential disease-modifying target for all three diseases.

## Supporting information

S1 File Supporting information contains all the supporting tables. (DOCX)
