# Supplementary material for: Biologic targets of prescription medications and risk of neurodegenerative disease in United States Medicare beneficiaries
Source: PLoS One. 2023 May 17;18(5):e0285011. doi: 10.1371/journal.pone.0285011 (PMC10191296; doi:10.1371/journal.pone.0285011)
Supplement: S1 File — (DOCX) [file pone.0285011.s001.docx]

**S1 Table. Target-action pairs with >10 beneficiaries in each group, U.S. Medicare 2009**

| **Target-action**^a^ **pair** | **PD only**  **N=28,679**  **n** | **AD only**  **N=8,332**  **n** | **ALS only**  **N=1,341**  **n** | **Mixed PD/AD/ALS**  **N=4,533**  **n** | **Controls**  **N=334,387**  **n** |
| --- | --- | --- | --- | --- | --- |
| 3-oxo-5-(alpha or beta)-steroid 4-dehydrogenase (blocker) | 1,368 | 261 | 50 | 184 | 9,867 |
| 5-hydroxytryptamine receptor (activator) | 4,125 | 1,821 | 113 | 1,086 | 22,130 |
| 5-hydroxytryptamine receptor (blocker) | 7,817 | 2,288 | 285 | 1,380 | 58,057 |
| 5-hydroxytryptamine receptor (other) | 1,466 | 183 | 31 | 184 | 6,140 |
| Acetylcholine receptor (activator) | 435 | 94 | 32 | 63 | 5,306 |
| Acetylcholine receptor (blocker) | 12,828 | 3,576 | 508 | 2,119 | 106,446 |
| Acetylcholine receptor (other) | 400 | 180 | 11 | 116 | 3,095 |
| Adenosine deaminase (blocker) | 492 | 127 | 16 | 116 | 3,302 |
| Adenosine receptor A (activator) | 3,809 | 1,081 | 162 | 625 | 32,343 |
| Adrenergic receptor (activator) | 9,566 | 2,803 | 415 | 1,530 | 92,228 |
| Adrenergic receptor (blocker) | 19,137 | 5,283 | 814 | 3,042 | 189,413 |
| Amyloid beta A4 protein (blocker) | 1,048 | 252 | 37 | 150 | 9,162 |
| Angiotensin-converting enzyme (blocker) | 9,984 | 3,024 | 416 | 1,653 | 113,412 |
| Angiotensin-converting enzyme (other) | 229 | 53 | 14 | 28 | 2,924 |
| Annexin (activator) | 4,933 | 1,311 | 198 | 709 | 49,180 |
| Antithrombin-III (activator) | 548 | 143 | 34 | 65 | 5,117 |
| Aquaporin (blocker) | 709 | 147 | 40 | 91 | 7,689 |
| Arachidonate 5-lipoxygenase (other) | 1,395 | 347 | 50 | 207 | 13,416 |
| Arginase-1 (other) | 533 | 147 | 22 | 92 | 3,907 |
| Aryl hydrocarbon receptor (activator) | 3,043 | 719 | 168 | 387 | 30,812 |
| ATP-binding cassette (ABC) transporter (activator) | 1,051 | 285 | 38 | 158 | 9,762 |
| ATP-binding cassette (ABC) transporter (blocker) | 1,845 | 529 | 52 | 328 | 17,239 |
| Atrial natriuretic peptide receptor 1 (activator) | 3,649 | 940 | 135 | 598 | 30,868 |
| B1 bradykinin receptor (other) | 1,181 | 291 | 52 | 172 | 11,701 |
| Bacterial outer membrane (blocker) | 2,037 | 485 | 83 | 312 | 19,270 |
| Beta-lactamase (blocker) | 2,626 | 703 | 129 | 406 | 26,432 |
| Bifunctional purine biosynthesis protein PURH (blocker) | 340 | 71 | 13 | 42 | 3,728 |
| Bile acids (other) | 496 | 137 | 17 | 88 | 4,417 |
| Calcitonin receptor (activator) | 569 | 156 | 26 | 80 | 4,492 |
| Calcium channel (blocker) | 11,829 | 3,399 | 520 | 1,908 | 117,137 |
| Calcium channel (other) | 1,399 | 311 | 63 | 194 | 9,904 |
| Calcium signal-modulating cyclophilin ligand (other) | 507 | 124 | 17 | 66 | 4,755 |
| Calcium/calmodulin-dependent 3',5'-cyclic nucleotide phosphodiesterase (blocker) | 429 | 140 | 25 | 65 | 5,045 |
| Calmodulin (blocker) | 2,249 | 619 | 82 | 387 | 19,504 |
| cAMP-specific 3',5'-cyclic phosphodiesterase (blocker) | 317 | 96 | 16 | 51 | 3,091 |
| Carbonic anhydrase (blocker) | 3,406 | 968 | 144 | 556 | 33,410 |
| cGMP-inhibited 3',5'-cyclic phosphodiesterase A (blocker) | 393 | 114 | 17 | 54 | 3,197 |
| cGMP-specific 3',5'-cyclic phosphodiesterase (blocker) | 158 | 19 | 13 | 19 | 1,911 |
| Cholinesterase (blocker) | 2,683 | 1,838 | 72 | 970 | 20,072 |
| Cytochrome P450 (CYP) (blocker) | 3,574 | 958 | 143 | 549 | 32,109 |
| Dihydrofolate reductase (blocker) | 4,014 | 1,201 | 143 | 618 | 36,780 |
| Dihydropteroate synthase/synthetase (blocker) | 4,183 | 1,235 | 151 | 649 | 38,230 |
| Dipeptidyl peptidase 4 (blocker) | 6,771 | 1,899 | 330 | 1,030 | 76,850 |
| DNA (other) | 695 | 202 | 27 | 95 | 4,962 |
| DNA gyrase (blocker) | 5,742 | 1,567 | 233 | 906 | 51,297 |
| DNA gyrase (other) | 6,897 | 1,982 | 260 | 1,075 | 64,543 |
| DNA polymerase (blocker) | 796 | 187 | 30 | 103 | 8,675 |
| DNA topoisomerase (blocker) | 4,155 | 1,085 | 181 | 634 | 40,519 |
| Dopamine receptor (activator) | 1,042 | 118 | 29 | 134 | 4,025 |
| Dopamine receptor (blocker) | 3,171 | 843 | 100 | 591 | 23,560 |
| Dopamine receptor (other) | 2,354 | 785 | 64 | 494 | 13,592 |
| Ergosterol (other) | 2,201 | 635 | 88 | 373 | 17,257 |
| Estrogen receptor (activator) | 3,102 | 887 | 123 | 450 | 35,058 |
| Evolved beta-galactosidase subunit alpha (other) | 902 | 277 | 27 | 178 | 4,994 |
| Extracellular calcium-sensing receptor (other) | 1,380 | 347 | 58 | 223 | 12,614 |
| Farnesyl pyrophosphate synthase (blocker) | 4,774 | 1,579 | 192 | 777 | 53,520 |
| Fe(II)-protoporphyrin IX (blocker) | 892 | 250 | 32 | 132 | 7,753 |
| Fibroblast growth factor (activator) | 647 | 150 | 29 | 96 | 4,714 |
| Folate receptor (other) | 428 | 133 | 16 | 76 | 3,486 |
| Gamma-aminobutyric acid receptor (activator) | 4,595 | 1,183 | 182 | 690 | 37,750 |
| Gamma-aminobutyric acid receptor (other) | 632 | 155 | 34 | 84 | 5,499 |
| Glucocorticoid receptor (activator) | 10,597 | 2,891 | 466 | 1,666 | 105,183 |
| Glucocorticoid receptor (blocker) | 936 | 188 | 43 | 93 | 8,617 |
| Glucocorticoid receptor (other) | 2,031 | 494 | 99 | 306 | 19,279 |
| Glutamate receptor (blocker) | 8,049 | 2,307 | 321 | 1,245 | 74,007 |
| Glycerol-3-phosphate dehydrogenase (blocker) | 4,243 | 1,094 | 153 | 649 | 44,283 |
| Group A nerve fibers (blocker) | 1,094 | 268 | 35 | 174 | 10,122 |
| Guanylate cyclase soluble subunit alpha-2 (activator) | 1,827 | 540 | 57 | 310 | 13,837 |
| HERG human cardiac K+ channel (blocker) | 1,434 | 379 | 58 | 214 | 12,157 |
| Histamine receptor (blocker) | 10,087 | 2,967 | 394 | 1,776 | 93,441 |
| Histamine receptor (other) | 1,438 | 444 | 49 | 275 | 8,683 |
| Histone deacetylase (blocker) | 7,845 | 2,189 | 331 | 1,222 | 86,412 |
| Hydroxyapatite (blocker) | 1,639 | 547 | 43 | 274 | 17,204 |
| Hypoxia-inducible factor 1-alpha (activator) | 1,854 | 560 | 74 | 272 | 17,641 |
| Insulin-degrading enzyme (blocker) | 306 | 68 | 14 | 56 | 2,560 |
| Insulin-like growth factor 1 receptor (other) | 2,590 | 740 | 78 | 435 | 19,481 |
| Integrin (blocker) | 3,029 | 841 | 140 | 477 | 35,018 |
| Integrin (other) | 5,413 | 1,593 | 218 | 844 | 57,680 |
| Isoleucine--tRNA ligase (blocker) | 1,409 | 412 | 62 | 236 | 12,109 |
| Lactoylglutathione lyase (blocker) | 536 | 106 | 29 | 72 | 6,512 |
| Lanosterol 14-alpha demethylase (blocker) | 411 | 108 | 28 | 58 | 3,670 |
| Lead chelators (other) | 1,648 | 547 | 43 | 274 | 17,292 |
| Leukotriene A-4 hydrolase (blocker) | 290 | 94 | 11 | 62 | 2,724 |
| Liver carboxylesterase 1 (other) | 5,072 | 1,454 | 186 | 775 | 45,386 |
| Lymphocyte antigen 96 (activator) | 343 | 93 | 17 | 47 | 2,471 |
| Matrix metalloproteinase (blocker) | 1,000 | 260 | 46 | 145 | 10,574 |
| Membrane primary amine oxidase (blocker) | 410 | 138 | 16 | 60 | 3,723 |
| Mineralocorticoid receptor (other) | 209 | 18 | 13 | 27 | 1,575 |
| Myeloperoxidase (blocker) | 560 | 177 | 24 | 109 | 5,983 |
| Neocarzinostatin (other) | 485 | 101 | 19 | 56 | 5,896 |
| Neurokinin 1 receptor (blocker) | 3,819 | 1,083 | 162 | 625 | 32,515 |
| Nicotinate-nucleotide pyrophosphorylase [carboxylating] (other) | 535 | 81 | 24 | 63 | 5,734 |
| Niemann-Pick C1-like protein 1 (blocker) | 3,184 | 822 | 175 | 435 | 38,519 |
| Nuclear receptor (activator) | 2,002 | 512 | 68 | 296 | 18,820 |
| Nuclear receptor (blocker) | 2,466 | 785 | 79 | 501 | 20,395 |
| Nuclear receptor (other) | 4,815 | 1,304 | 189 | 706 | 41,706 |
| Opioid receptor (activator) | 15,481 | 4,270 | 701 | 2,340 | 153,878 |
| Opioid receptor (blocker) | 192 | 34 | 12 | 23 | 1,614 |
| Oxygen-insensitive NADH/NAD(P)H nitroreductase (activator) | 3,963 | 1,142 | 146 | 646 | 36,220 |
| P-glycoprotein 1 (blocker) | 1,042 | 297 | 39 | 168 | 12,802 |
| P2Y purinoceptor 12 (blocker) | 4,009 | 1,236 | 159 | 661 | 33,736 |
| Penicillin-binding protein (blocker) | 12,051 | 3,211 | 539 | 1,760 | 127,482 |
| Peptostreptococcal albumin-binding protein (other) | 1,903 | 573 | 83 | 312 | 22,092 |
| Peroxisome proliferator-activated receptor (activator) | 2,226 | 635 | 72 | 341 | 22,195 |
| Peroxisome proliferator-activated receptor (other) | 1,367 | 357 | 53 | 187 | 12,461 |
| Potassium channel (blocker) | 4,177 | 1,138 | 176 | 694 | 38,366 |
| Potassium-transporting ATPase alpha chain 1 (blocker) | 10,117 | 2,734 | 395 | 1,563 | 89,939 |
| Progesterone receptor (activator) | 4,441 | 1,256 | 200 | 697 | 45,041 |
| Prostaglandin receptor (activator) | 2,366 | 751 | 96 | 445 | 24,306 |
| Prostaglandin synthase (blocker) | 16,643 | 4,594 | 741 | 2,496 | 171,971 |
| Protein S100 (calcium binding cytosolic protein family) (blocker) | 954 | 250 | 45 | 143 | 8,869 |
| Retinal rhodopsin-sensitive cGMP 3',5'-cyclic phosphodiesterase subunit gamma (blocker) | 454 | 60 | 22 | 58 | 5,228 |
| Retinoic acid receptor (activator) | 506 | 115 | 14 | 74 | 3,910 |
| Ribosomal protein (blocker) | 2,044 | 492 | 98 | 294 | 20,338 |
| Ribosomal protein (other) | 453 | 146 | 20 | 73 | 3,425 |
| Ribosomal RNA (blocker) | 7,844 | 2,049 | 342 | 1,145 | 81,644 |
| Ribosomal RNA (other) | 2,234 | 549 | 102 | 296 | 22,244 |
| Sigma receptor (blocker) | 2,084 | 741 | 77 | 380 | 15,147 |
| Sodium channel (blocker) | 9,056 | 2,531 | 363 | 1,417 | 82,248 |
| Sodium-dependent dopamine transporter (blocker) | 2,392 | 539 | 76 | 392 | 12,563 |
| Sodium-dependent noradrenaline transporter (blocker) | 4,806 | 1,320 | 201 | 786 | 43,324 |
| Sodium-dependent serotonin transporter (blocker) | 6,822 | 1,967 | 270 | 1,118 | 52,986 |
| Sodium/potassium-transporting ATPase subunit alpha-1 (blocker) | 2,681 | 742 | 119 | 422 | 24,515 |
| Sodium/potassium-transporting ATPase subunit alpha-1 (other) | 7,659 | 2,230 | 304 | 1,264 | 71,360 |
| Solute carrier (SLC) (blocker) | 19,511 | 5,934 | 830 | 3,199 | 205,133 |
| Solute carrier (SLC) (other) | 1,547 | 462 | 71 | 250 | 15,928 |
| Steroid 21-hydroxylase (blocker) | 1,023 | 261 | 35 | 154 | 7,797 |
| Sterol regulatory element-binding protein 1 (blocker) | 232 | 57 | 15 | 24 | 2,314 |
| Thymidine kinase (activator) | 1,162 | 277 | 58 | 174 | 12,916 |
| Thymidylate synthase (other) | 355 | 73 | 15 | 48 | 3,935 |
| Toll-like receptor (TLR) (activator) | 374 | 94 | 15 | 59 | 2,907 |
| Toll-like receptor (TLR) (blocker) | 1,917 | 461 | 102 | 273 | 19,964 |
| Transient receptor potential (TRP) channel (activator) | 3,809 | 1,081 | 162 | 625 | 32,344 |
| Transient receptor potential (TRP) channel (blocker) | 1,642 | 478 | 47 | 253 | 15,713 |
| Tubulin alpha/beta chain (blocker) | 727 | 165 | 24 | 105 | 7,470 |
| Type-1 angiotensin II receptor (blocker) | 4,761 | 1,336 | 216 | 723 | 53,784 |
| Vascular endothelial growth factor A (other) | 1,536 | 447 | 59 | 222 | 14,696 |
| Vascular endothelial growth factor receptor (blocker) | 410 | 118 | 15 | 70 | 3,532 |
| Vitamin D3 receptor (activator) | 517 | 166 | 23 | 89 | 5,705 |
| Vitamin D3 receptor (blocker) | 302 | 96 | 11 | 51 | 2,997 |
| Xanthine dehydrogenase/oxidase (blocker) | 1,294 | 267 | 58 | 159 | 13,116 |

^a^ We classified each action into the closest of one of the following three categories: activator, blocker, or other.

**S2 Table. Medication target-action pairs inversely associated with neurodegenerative condition(s) (mean OR<1.00) but <20% average reduction in relative risk,** **by significance, U.S. Medicare 2009**

| **Target (action)**  **Medication(s)** | | **Adjusted OR (95% CI)^a^** | | | |  |
| --- | --- | --- | --- | --- | --- | --- |
|  |  | **PD only**  **N=28,679** | **AD only**  **N=8,332** | **ALS only**  **N=1,341** | **Mixed PD/AD/**  **ALS**  **N=4,533** | **Mean OR** |
| **Significant association(s)** |  |  |  |  |  |  |
| Lactoylglutathione lyase (blocker) | Indomethacin | **0.84 (0.77, 0.92)** | **0.67 (0.55, 0.82)** | 0.97 (0.67, 1.41) | **0.76 (0.60, 0.96)** | 0.81 |
| Hypoxia-inducible factor 1-alpha (activator) | Carvedilol, hydralazine | **0.82 (0.78, 0.87)** | 0.92 (0.84, 1.01) | 0.82 (0.64, 1.04) | **0.73 (0.64, 0.83)** | 0.82 |
| Nicotinate-nucleotide pyrophosphorylase [carboxylating] (other) | Niacin | 0.95 (0.87, 1.04) | **0.68 (0.54, 0.84)** | 0.88 (0.59, 1.33) | 0.82 (0.64, 1.05) | 0.83 |
| Retinal rhodopsin-sensitive cGMP 3',5'-cyclic phosphodiesterase subunit gamma (blocker) | Sildenafil | 0.91 (0.83, 1.01) | **0.70 (0.54, 0.91)** | 0.81 (0.53, 1.25) | 0.91 (0.70, 1.19) | 0.83 |
| Neocarzinostatin (other) | Testosterone succinate | 0.99 (0.90, 1.09) | **0.81 (0.66, 0.99)** | 0.80 (0.51, 1.26) | 0.79 (0.61, 1.04) | 0.85 |
| Angiotensin-converting enzyme (other) | Chloroquine, hydroxychloroquine | **0.84 (0.73, 0.96)** | 0.78 (0.59, 1.02) | 1.06 (0.63, 1.80) | 0.73 (0.51, 1.07) | 0.85 |
| Nuclear receptor (other) | Atorvastatin, cyclophosphamide, dasatinib, docetaxel, flutamide, ketoconazole, lindane, spironolactone, tamoxifen, warfarin | **0.88 (0.85, 0.91)** | **0.91 (0.86, 0.97)** | **0.84 (0.72, 0.97)** | **0.80 (0.74, 0.87)** | 0.86 |
| Semicarbazide-sensitive amine oxidase (blocker) | Hydralazine | **0.88 (0.79, 0.97)** | 0.98 (0.82, 1.17) | 0.86 (0.52, 1.41) | **0.74 (0.57, 0.96)** | 0.87 |
| Vitamin D3 receptor (blocker) | Calcipotriol, calcitriol, doxercalciferol | **0.86 (0.76, 0.96)** | 1.10 (0.90, 1.35) | 0.68 (0.38, 1.23) | 0.98 (0.75, 1.29) | 0.91 |
| Niemann-Pick C1-like protein 1 (blocker) | Ezetimibe | **0.88 (0.85, 0.92)** | **0.90 (0.84, 0.97)** | 1.06 (0.90, 1.25) | **0.82 (0.75, 0.91)** | 0.92 |
| Antithrombin-III (activator) | Dalteparin, enoxaparin, fondaparinux, heparin, tinzaparin | **0.85 (0.77, 0.93)** | 0.94 (0.80, 1.12) | 1.20 (0.85, 1.70) | **0.69 (0.54, 0.89)** | 0.92 |
| Type-1 angiotensin II receptor (blocker) | Azilsartan medoxomil, candesartan cilexetil, eprosartan, irbesartan, olmesartan, telmisartan, valsartan | **0.95 (0.92, 0.98)** | **0.88 (0.82, 0.93)** | 0.98 (0.85, 1.14) | **0.88 (0.81, 0.95)** | 0.92 |
| Sodium/potassium-transporting ATPase subunit alpha-1 (blocker) | Bisacodyl, digoxin, etacrynic acid | **0.91 (0.87, 0.95)** | **0.91 (0.84, 0.98)** | 1.00 (0.82, 1.21) | **0.89 (0.80, 0.99)** | 0.93 |
| Peroxisome proliferator-activated receptor (activator) | Amiodarone, balsalazide, fenofibrate, fenoprofen, gemfibrozil, glipizide, ibuprofen, indomethacin, mesalazine, nateglinide, omega-3 fatty acids, pioglitazone, repaglinide, rosiglitazone, sulfasalazine, telmisartan | 1.00 (0.96, 1.04) | 1.05 (0.97, 1.13) | **0.73 (0.58, 0.92)** | 0.99 (0.89, 1.09) | 0.94 |
| Glucocorticoid receptor (blocker) | Budesonide, spironolactone | 1.11 (1.03, 1.19) | 0.87 (0.75, 1.01) | 1.05 (0.78, 1.43) | **0.75 (0.61, 0.92)** | 0.95 |
| Transient receptor potential (TRP) channel (blocker) | Butamben, glyburide | 1.04 (0.98, 1.09) | 1.10 (1.00, 1.21) | **0.69 (0.51, 0.92)** | 0.99 (0.87, 1.13) | 0.96 |
| Angiotensin-converting enzyme (blocker) | Benazepril, captopril, enalapril, fosinopril, lisinopril, moexipril, perindopril, quinapril, ramipril, trandolapril | **0.96 (0.93, 0.98)** | 1.03 (0.99, 1.07) | **0.84 (0.76, 0.94)** | 1.00 (0.94, 1.06) | 0.96 |
| Aryl hydrocarbon receptor (activator) | Atorvastatin, flutamide, leflunomide, omeprazole, nimodipine | **0.95 (0.91, 0.99)** | 0.92 (0.85, 1.00) | 1.13 (0.96, 1.34) | **0.84 (0.75, 0.93)** | 0.96 |
| Atrial natriuretic peptide receptor 1 (activator) | Isosorbide dinitrate, nitroglycerin | 1.01 (0.97, 1.04) | **0.93 (0.87, 1.00)**^b^ | 0.88 (0.74, 1.05) | 1.04 (0.96, 1.13) | 0.97 |
| Prostaglandin receptor (activator) | Alprostadil, bimatoprost, latanoprost, misoprostol, travoprost | 0.96 (0.93, 1.00) | **0.91 (0.85, 0.97)** | 0.94 (0.78, 1.12) | 1.05 (0.97, 1.14) | 0.97 |
| Dipeptidyl peptidase 4 (blocker) | Atorvastatin, sitagliptin | **0.95 (0.92, 0.97)** | 0.98 (0.93, 1.03) | 1.02 (0.90, 1.16) | **0.92 (0.86, 0.99)** | 0.97 |
| Lead chelators (other) | Edetate disodium anhydrous, succimer | 1.09 (1.04, 1.15) | 1.06 (0.97, 1.16) | **0.67 (0.49, 0.91)** | 1.06 (0.94, 1.21) | 0.97 |
| ATP-binding cassette (ABC) transporter (blocker) | Chlorpropamide, glipizide, glyburide, nateglinide, repaglinide, tolbutamide | 1.04 (0.98, 1.09) | 1.06 (0.97, 1.16) | **0.67 (0.51, 0.89)** | 1.14 (1.01, 1.28) | 0.98 |
| Nuclear receptor (activator) | Carbamazepine, clonazepam, clotrimazole, dexamethasone, erlotinib, ethinylestradiol, fenofibrate, mephenytoin, nifedipine, oxiconazole, paclitaxel, phenobarbital, rifampicin, rifaximin, ritonavir | 1.08 (1.03, 1.13) | 1.01 (0.92, 1.10) | **0.78 (0.61, 1.00)**^b^ | 1.05 (0.93, 1.18) | 0.98 |
| Hydroxyapatite (blocker) | Ibandronate, risedronic acid, zoledronic acid | 1.09 (1.04, 1.16) | 1.07 (0.97, 1.17) | **0.67 (0.49, 0.91)** | 1.07 (0.94, 1.21) | 0.98 |
| Glycerol-3-phosphate dehydrogenase (blocker) | Metformin | 1.04 (1.01, 1.08) | 1.03 (0.96, 1.10) | **0.80 (0.67, 0.95)** | 1.06 (0.97, 1.15) | 0.98 |
|  | | | | | | |
| **No significant associations** |  |  |  |  |  |  |
| Thymidylate synthase (other) | Fluorouracil | 0.91 (0.82, 1.02) | 0.81 (0.64, 1.02) | 0.79 (0.48, 1.32) | 0.87 (0.65, 1.16) | 0.85 |
| DNA polymerase (blocker) | Acyclovir, azelaic acid, famciclovir, ganciclovir, penciclovir, tenofovir disoproxil, valacyclovir | 1.01 (0.93, 1.08) | 0.90 (0.77, 1.04) | 0.78 (0.54, 1.12) | 0.89 (0.73, 1.09) | 0.90 |
| P-glycoprotein 1 (blocker) | Medroxyprogesterone acetate, verapamil | 0.96 (0.89, 1.02) | 0.91 (0.81, 1.03) | 0.75 (0.55, 1.03) | 0.99 (0.85, 1.16) | 0.90 |
| B1 bradykinin receptor (other) | Captopril, ramipril | 0.97 (0.91, 1.03) | 0.90 (0.80, 1.02) | 0.97 (0.74, 1.29) | 0.91 (0.78, 1.06) | 0.94 |
| cGMP-specific 3',5'-cyclic phosphodiesterase (blocker) | Dipyridamole, sildenafil, tadalafil, theophylline, vardenafil | 0.90 (0.76, 1.06) | 0.67 (0.43, 1.06) | 1.33 (0.77, 2.31) | 0.88 (0.56, 1.39) | 0.95 |
| ATP-binding cassette (ABC) transporter (activator) | Glimepiride | 1.00 (0.93, 1.07) | 1.00 (0.89, 1.13) | 0.86 (0.62, 1.19) | 0.94 (0.80, 1.11) | 0.95 |
| Histone deacetylase (blocker) | Atorvastatin, fluvastatin, pravastatin, simvastatin, valproic acid | 0.97 (0.95, 1.00) | 0.98 (0.94, 1.03) | 0.90 (0.80, 1.01) | 0.99 (0.93, 1.06) | 0.96 |
| Calcium signal-modulating cyclophilin ligand (other) | Cyclosporine | 1.13 (1.03, 1.24) | 0.96 (0.80, 1.15) | 0.84 (0.52, 1.36) | 0.95 (0.74, 1.21) | 0.97 |
| Calcium/calmodulin-dependent 3',5'-cyclic nucleotide phosphodiesterase (blocker) | Felodipine, nicardipine | 0.91 (0.82, 1.00) | 0.95 (0.80, 1.12) | 1.21 (0.81, 1.79) | 0.82 (0.64, 1.05) | 0.97 |
| Ribosomal RNA (blocker) | Azithromycin, cethromycin, chlortetracycline, erythromycin, linezolid, minocycline, neomycin, paromomycin, telithromycin, tetracycline, tobramycin | 1.03 (1.01, 1.06) | 0.96 (0.91, 1.01) | 0.95 (0.83, 1.07) | 0.97 (0.91, 1.04) | 0.98 |
| Ribosomal RNA (other) | Doxycycline, gentamicin, tigecycline | 1.03 (0.99, 1.08) | 0.99 (0.90, 1.08) | 0.98 (0.80, 1.20) | 0.93 (0.82, 1.05) | 0.98 |
| Penicillin-binding protein (blocker) | Amoxicillin, ampicillin, aztreonam, benzylpenicillin, carbenicillin, cefaclor, cefadroxil, cefazolin, cefdinir, cefditoren, cefixime, cefotaxime, cefotetan, cefoxitin, cefprozil, ceftazidime, ceftibuten, ceftriaxone, cefuroxime, cephalexin, cloxacillin, dicloxacillin, doripenem, ertapenem, imipenem, nafcillin, piperacillin, phenoxymethylpenicillin | 1.02 (0.99, 1.04) | 0.98 (0.94, 1.03) | 0.96 (0.86, 1.07) | 0.94 (0.88, 1.00) | 0.98 |
| Arachidonate 5-lipoxygenase (other) | Montelukast | 1.09 (1.03, 1.16) | 0.99 (0.89, 1.11) | 0.80 (0.60, 1.06) | 1.07 (0.93, 1.24) | 0.99 |
| Annexin (activator) | Amcinonide, clobetasol propionate, dexamethasone, fluocinolone acetonide, hydrocortisone, methylprednisolone | 1.05 (1.02, 1.08) | 1.04 (0.99, 1.10) | 0.89 (0.78, 1.02) | 0.98 (0.91, 1.05) | 0.99 |
| DNA topoisomerase (blocker) | Ciprofloxacin, gatifloxacin, gemifloxacin, levofloxacin, moxifloxacin, norfloxacin, ofloxacin, podofilox | 1.03 (1.00, 1.06) | 0.95 (0.90, 1.01) | 0.99 (0.85, 1.15) | 1.00 (0.92, 1.08) | 0.99 |
| Thymidine kinase (activator) | Acyclovir, ganciclovir, penciclovir, valacyclovir | 0.99 (0.93, 1.06) | 0.92 (0.81, 1.04) | 1.02 (0.78, 1.33) | 1.04 (0.89, 1.21) | 0.99 |
| ^a^ OR comparing those who did vs. did not use any of the listed medications prior to diagnosis/reference, adjusted for age (continuous, using Harrell’s method), sex, race/ethnicity (4 categories: White, Black, Hispanic, and API/other/unknown), use of medical care (total number of physician/outpatient visits, total number of days in a hospital/skilled nursing facility, and total number of medical conditions, each prior the case diagnosis or control reference, as a continuous variable), and a dichotomous indicator of smoking (lung cancer or chronic obstructive pulmonary disease).  ^b^ p<0.05 (upper CI excludes 1.00 before rounding).  Abbreviations: AD=Alzheimer disease; ALS=amyotrophic lateral sclerosis; API=Asian/Pacific Islander; CI=confidence interval; OR=odds ratio; PD=Parkinson disease. | | | | | | |

**S3 Table. Medication target-action pairs not inversely associated with neurodegenerative conditions (mean OR ≥1.00), U.S. Medicare 2009**

|  |  | **Adjusted OR (95% CI)**^a^ | | | |  |
| --- | --- | --- | --- | --- | --- | --- |
| **Target (action)** | **Medication(s)**^b^ | **PD only**  **N=28,679** | **AD only**  **N=8,332** | **ALS only**  **N=1,341** | **Mixed PD/AD/**  **ALS**  **N=4,533** | **Mean OR** |
| Guanylate cyclase soluble subunit alpha-2 (activator) | Isosorbide mononitrate | 1.05 (1.00, 1.11) | 1.06 (0.97, 1.17) | 0.82 (0.62, 1.07) | 1.05 (0.94, 1.19) | 1.00 |
| Integrin (other) | Efalizumab, levothyroxine | 1.06 (1.02, 1.09) | 0.97 (0.92, 1.03) | 0.94 (0.81, 1.09) | 1.01 (0.94, 1.10) | 1.00 |
| Integrin (blocker) | Lovastatin, rosuvastatin, simvastatin, tinzaparin | 0.99 (0.95, 1.03) | 1.01 (0.94, 1.09) | 0.97 (0.82, 1.15) | 1.04 (0.95, 1.14) | 1.00 |
| Vascular endothelial growth factor receptor (blocker) | Sorafenib, sunitinib | 0.98 (0.88, 1.09) | 1.09 (0.90, 1.31) | 0.86 (0.51, 1.43) | 1.08 (0.85, 1.38) | 1.00 |
| Aquaporin (blocker) | Acetazolamide, bisacodyl, sennosides | 1.01 (0.93, 1.09) | 0.87 (0.74, 1.03) | 1.17 (0.85, 1.61) | 0.93 (0.76, 1.15) | 1.00 |
| Ribosomal protein (blocker) | Amikacin, chlortetracycline, clarithromycin, clindamycin, demeclocycline, lincomycin, minocycline, neomycin, nitrofurantoin, paromomycin, retapamulin, spiramycin, tetracycline, tobramycin | 1.03 (0.98, 1.08) | 0.95 (0.86, 1.04) | 1.06 (0.86, 1.31) | 1.00 (0.89, 1.13) | 1.01 |
| Opioid receptor (blocker) | Buprenorphine, butorphanol, lamotrigine, naltrexone, oxymorphone, pentazocine | 1.00 (0.85, 1.16) | 0.82 (0.58, 1.16) | 1.29 (0.73, 2.28) | 0.92 (0.61, 1.39) | 1.01 |
| Estrogen receptor (activator) | Conjugated estrogens, danazol, estrone sulfate, ethinylestradiol, medroxyprogesterone acetate, mestranol, norgestimate, raloxifene | 1.09 (1.05, 1.13) | 1.02 (0.95, 1.09) | 0.90 (0.76, 1.08) | 1.04 (0.94, 1.14) | 1.01 |
| Fe(II)-protoporphyrin IX (blocker) | Quinine | 1.10 (1.02, 1.18) | 1.04 (0.92, 1.19) | 0.91 (0.64, 1.30) | 1.00 (0.84, 1.20) | 1.01 |
| Matrix metalloproteinase (blocker) | Captopril, fenofibrate, minocycline | 0.98 (0.92, 1.05) | 1.09 (0.96, 1.23) | 0.98 (0.73, 1.32) | 1.00 (0.85, 1.19) | 1.01 |
| Sodium/potassium-transporting ATPase subunit alpha-1 (other) | Aluminum acetate, ciclopirox, potassium | 1.05 (1.02, 1.08) | 1.01 (0.96, 1.06) | 0.93 (0.82, 1.06) | 1.06 (0.99, 1.13) | 1.01 |
| Farnesyl pyrophosphate synthase (blocker) | Alendronic acid, ibandronate, risedronic acid, zoledronic acid | 1.05 (1.01, 1.09) | 1.01 (0.95, 1.07) | 0.99 (0.84, 1.16) | 1.00 (0.92, 1.08) | 1.01 |
| Progesterone receptor (activator) | Danazol, fluticasone, medroxyprogesterone acetate, meprednisone, norgestimate, progesterone, spironolactone | 1.01 (0.98, 1.05) | 1.05 (0.98, 1.12) | 0.96 (0.82, 1.12) | 1.03 (0.95, 1.13) | 1.01 |
| Amyloid beta A4 protein (blocker) | Tromethamine | 1.14 (1.07, 1.22) | 0.97 (0.85, 1.10) | 0.93 (0.67, 1.30) | 1.02 (0.87, 1.21) | 1.02 |
| cAMP-specific 3',5'-cyclic phosphodiesterase (blocker) | Caffeine, dipyridamole, dyphylline, papaverine, theophylline | 0.98 (0.87, 1.10) | 1.08 (0.88, 1.33) | 1.04 (0.63, 1.71) | 0.99 (0.75, 1.32) | 1.02 |
| cGMP-inhibited 3',5'-cyclic phosphodiesterase A (blocker) | Aminophylline, anagrelide, cilostazol, theophylline | 1.06 (0.95, 1.18) | 1.08 (0.89, 1.31) | 1.10 (0.68, 1.78) | 0.87 (0.66, 1.14) | 1.03 |
| Carbonic anhydrase (blocker) | Acetazolamide, amlodipine, bendroflumethiazide, brinzolamide, celecoxib, chlorothiazide, chlorthalidone, dorzolamide, furosemide, methazolamide, methocarbamol, methyclothiazide, topiramate, zonisamide | 1.06 (1.02, 1.10) | 1.02 (0.96, 1.08) | 1.05 (0.90, 1.23) | 1.04 (0.95, 1.12) | 1.04 |
| Group A nerve fibers (blocker) | Phenazopyridine | 1.15 (1.08, 1.23) | 1.02 (0.90, 1.16) | 0.78 (0.55, 1.09) | 1.22 (1.05, 1.43) | 1.04 |
| Peptostreptococcal albumin-binding protein (other) | Naproxen | 1.03 (0.98, 1.09) | 1.10 (1.01, 1.20) | 0.95 (0.76, 1.18) | 1.09 (0.97, 1.22) | 1.04 |
| Peroxisome proliferator-activated receptor (other) | Fenoprofen, omega-3 fatty acids, rosiglitazone, valproic acid | 1.11 (1.05, 1.18) | 1.10 (0.99, 1.23) | 0.99 (0.75, 1.30) | 0.97 (0.83, 1.12) | 1.04 |
| Solute carrier (SLC) (other) | Ampicillin, benzoic acid, cefotaxime, ceftriaxone, clarithromycin, levocarnitine | 1.00 (0.95, 1.06) | 1.14 (1.04, 1.25) | 0.98 (0.78, 1.24) | 1.05 (0.92, 1.19) | 1.04 |
| Calcium channel (blocker) | Amiodarone, amlodipine, butamben, diltiazem, ethosuximide, felodipine, gabapentin, isradipine, lamotrigine, levetiracetam, magnesium sulfate, nicardipine, nifedipine, nimodipine, nisoldipine, phenytoin, ranolazine, spironolactone, topiramate, verapamil, zonisamide | 1.07 (1.04, 1.10) | 0.98 (0.94, 1.03) | 1.07 (0.96, 1.20) | 1.04 (0.98, 1.11) | 1.04 |
| Acetylcholine receptor (activator) | Bethanechol, cevimeline, metoclopramide, nicotine, pilocarpine, varenicline | 0.97 (0.89, 1.07) | 0.93 (0.77, 1.14) | 1.26 (0.89, 1.77) | 1.04 (0.82, 1.33) | 1.05 |
| Vitamin D3 receptor (activator) | Cholecalciferol, ergocalciferol, paricalcitol | 1.03 (0.94, 1.13) | 1.02 (0.87, 1.19) | 1.08 (0.72, 1.64) | 1.08 (0.87, 1.33) | 1.05 |
| Prostaglandin synthase (blocker) | Acetaminophen, antipyrine, aspirin, balsalazide, bromfenac, celecoxib, diclofenac, diflunisal, etodolac, fenoprofen, flurbiprofen, ibuprofen, indomethacin, ketoprofen, ketorolac, lenalidomide, magnesium salicylate, mefenamic acid, meloxicam, mesalazine, nabumetone, naproxen, nepafenac, nitroaspirin, oxaprozin, phenyl salicylate, piroxicam, salicylic acid, salsalate, sulfasalazine, sulindac, thalidomide, tolmetin | 1.10 (1.08, 1.13) | 1.05 (1.00, 1.10) | 1.03 (0.92, 1.15) | 1.00 (0.94, 1.07) | 1.05 |
| Beta-lactamase (blocker) | Citric acid, clavulanic acid, sulbactam, tazobactam | 1.01 (0.96, 1.05) | 1.06 (0.98, 1.15) | 1.05 (0.87, 1.26) | 1.07 (0.96, 1.18) | 1.05 |
| DNA gyrase (blocker) | Ciprofloxacin, gatifloxacin, gemifloxacin, levofloxacin, moxifloxacin, norfloxacin, ofloxacin | 1.08 (1.05, 1.12) | 1.09 (1.03, 1.15) | 0.94 (0.81, 1.09) | 1.11 (1.03, 1.20) | 1.06 |
| Solute carrier (SLC) (blocker) | Bendroflumethiazide, benzylpenicillin, bumetanide, chlorothiazide, chlorthalidone, etacrynic acid, furosemide, gemfibrozil, hydrochlorothiazide, indapamide, methyclothiazide, metolazone, probenecid, torasemide | 1.07 (1.05, 1.09) | 1.09 (1.06, 1.12) | 0.97 (0.89, 1.04) | 1.09 (1.05, 1.13) | 1.06 |
| Toll-like receptor (TLR) (activator) | Imiquimod, papain | 1.09 (0.98, 1.22) | 1.04 (0.84, 1.28) | 1.00 (0.60, 1.67) | 1.09 (0.84, 1.42) | 1.06 |
| Myeloperoxidase (blocker) | Cefdinir | 0.94 (0.86, 1.02) | 1.18 (1.01, 1.38) | 0.84 (0.56, 1.27) | 1.28 (1.05, 1.55) | 1.06 |
| DNA gyrase (other) | Ciprofloxacin | 1.12 (1.09, 1.16) | 1.15 (1.09, 1.21) | 0.88 (0.77, 1.02) | 1.12 (1.05, 1.20) | 1.07 |
| P2Y purinoceptor 12 (blocker) | Clopidogrel, ticlopidine | 1.04 (1.00, 1.08) | 1.19 (1.12, 1.27) | 1.00 (0.84, 1.18) | 1.06 (0.98, 1.16) | 1.07 |
| Liver carboxylesterase 1 (other) | Dextropropoxyphene, levocarnitine, meperidine | 1.16 (1.12, 1.20) | 1.10 (1.03, 1.16) | 0.93 (0.79, 1.09) | 1.09 (1.01, 1.18) | 1.07 |
| Dihydrofolate reductase (blocker) | Methotrexate, pyrimethamine, trimethoprim | 1.14 (1.10, 1.18) | 1.20 (1.13, 1.28) | 0.85 (0.71, 1.02) | 1.11 (1.02, 1.21) | 1.08 |
| Bacterial outer membrane (blocker) | Chlorhexidine, colistimethate, colistin, polymyxin B | 1.14 (1.09, 1.19) | 1.00 (0.91, 1.09) | 1.03 (0.83, 1.28) | 1.14 (1.02, 1.28) | 1.08 |
| Sterol regulatory element-binding protein 1 (blocker) | Omega-3 fatty acids | 1.02 (0.89, 1.18) | 1.12 (0.86, 1.47) | 1.41 (0.85, 2.36) | 0.78 (0.52, 1.16) | 1.08 |
| Glucocorticoid receptor (activator) | Alclometasone, amcinonide, beclomethasone dipropionate, betamethasone, clobetasol propionate, cortisone acetate, desonide, desoximetasone, dexamethasone, diflorasone, flunisolide, fluocinolone acetonide, fluorometholone, flurandrenolide, fluticasone, hydrocortisone, loteprednol etabonate, meprednisone, methylprednisolone, prednicarbate, prednisolone, progesterone, rimexolone, triamcinolone | 1.10 (1.07, 1.13) | 1.05 (1.00, 1.10) | 1.04 (0.92, 1.16) | 1.11 (1.04, 1.18) | 1.08 |
| 3-oxo-5-(alpha or beta)-steroid 4-dehydrogenase (blocker) | Azelaic acid, dutasteride, finasteride, levonorgestrel, norgestrel, spironolactone | 1.16 (1.10, 1.23) | 1.12 (0.99, 1.26) | 0.99 (0.77, 1.29) | 1.07 (0.93, 1.23) | 1.09 |
| Oxygen-insensitive NADH/NAD(P)H nitroreductase (activator) | Metronidazole, nitrofurantoin | 1.14 (1.11, 1.18) | 1.12 (1.05, 1.18) | 0.92 (0.78, 1.08) | 1.17 (1.09, 1.27) | 1.09 |
| Adrenergic receptor (activator)^b^ | Acebutolol, albuterol, amphetamine, apraclonidine, arformoterol, bambuterol, brimonidine, bromocriptine, buspirone, carteolol, clenbuterol, clonidine, dihydroergotamine, dipivefrin, epinephrine, ergotamine, fenoterol, guanabenz, levosalbutamol, midodrine, modafinil, naphazoline, paliperidone, phenylephrine, pirbuterol, pramipexole, pseudoephedrine, salmeterol, terbutaline, tizanidine | 1.10 (1.07, 1.13) | 1.12 (1.07, 1.18) | 1.03 (0.91, 1.16) | 1.11 (1.04, 1.18) | 1.09 |
| Dihydropteroate synthase/synthetase (blocker) | Acetyl sulfisoxazole, sulfacetamide, sulfadiazine, sulfamethoxazole, sulfanilamide | 1.14 (1.10, 1.18) | 1.19 (1.12, 1.26) | 0.91 (0.77, 1.07) | 1.12 (1.03, 1.22) | 1.09 |
| Toll-like receptor (TLR) (blocker) | Adapalene, chloroquine, cyclobenzaprine, hydroxychloroquine | 1.08 (1.03, 1.14) | 1.02 (0.92, 1.12) | 1.18 (0.96, 1.45) | 1.08 (0.96, 1.23) | 1.09 |
| Opioid receptor (activator) | Amitriptyline, atomoxetine, buprenorphine, butorphanol, codeine, dextromethorphan, dextropropoxyphene, dihydromorphine, diphenoxylate, fentanyl, hydrocodone, meperidine, mirtazapine, morphine, oxycodone, oxymorphone, pentazocine, progesterone, tramadol | 1.13 (1.10, 1.16) | 1.07 (1.03, 1.13) | 1.11 (0.99, 1.24) | 1.05 (0.99, 1.12) | 1.09 |
| Potassium channel (blocker) | Amitriptyline, atomoxetine, butamben, carvedilol, chlorpromazine, clotrimazole, dofetilide, erythromycin, flecainide, fluoxetine, glimepiride, glyburide, hydrochlorothiazide, hydroxyzine, imipramine, ketoconazole, loratadine, nefazodone, nifedipine, phenytoin, pimozide, procainamide, propafenone, quinidine, quinine, ranolazine, sotalol, tamoxifen, terazosin, thioridazine, tolbutamide, verapamil | 1.09 (1.05, 1.13) | 1.09 (1.02, 1.16) | 1.01 (0.86, 1.19) | 1.17 (1.08, 1.27) | 1.09 |
| Cytochrome P450 (CYP) (blocker) | Anastrozole, clotrimazole, exemestane, fluconazole, letrozole, nicotine, oxiconazole, posaconazole, sertaconazole, spironolactone, terconazole, voriconazole | 1.15 (1.11, 1.18) | 1.11 (1.04, 1.18) | 0.98 (0.84, 1.14) | 1.16 (1.07, 1.25) | 1.10 |
| Bile acids (other) | Cholestyramine, colesevelam | 1.10 (1.00, 1.21) | 1.13 (0.96, 1.34) | 0.88 (0.56, 1.39) | 1.29 (1.05, 1.59) | 1.10 |
| Glutamate receptor (blocker) | Acamprosate, amantadine, atomoxetine, butabarbital, butalbital, dextromethorphan, dextropropoxyphene, felbamate, guaifenesin, haloperidol, lamotrigine, magnesium carbonate, meperidine, orphenadrine, phenobarbital, primidone, tramadol | 1.17 (1.13, 1.20) | 1.11 (1.06, 1.17) | 1.00 (0.88, 1.14) | 1.11 (1.04, 1.19) | 1.10 |
| Extracellular calcium-sensing receptor (other) | Neomycin | 1.15 (1.08, 1.22) | 1.03 (0.93, 1.15) | 1.07 (0.82, 1.39) | 1.19 (1.04, 1.36) | 1.11 |
| Glucocorticoid receptor (other) | Hydrocortisone acetate, levonorgestrel, mometasone furoate | 1.12 (1.07, 1.18) | 1.03 (0.93, 1.13) | 1.16 (0.94, 1.43) | 1.13 (1.00, 1.27) | 1.11 |
| Adrenergic receptor (blocker)^b^ | Alfuzosin, amiodarone, amitriptyline, amoxapine, aripiprazole, atenolol, betaxolol, bisoprolol, cabergoline, carteolol, carvedilol, chlorpromazine, clozapine, desipramine, dextroamphetamine, doxazosin, droperidol, epinephrine, escitalopram, esmirtazapine, imipramine, lamotrigine, levobunolol, maprotiline, metipranolol, metoprolol, mirtazapine, nadolol, nefazodone, nicardipine, nortriptyline, olanzapine, paliperidone, paroxetine, perospirone, phenoxybenzamine, phentolamine, prochlorperazine, promethazine, propafenone, propranolol, quetiapine, quinidine, ranolazine, risperidone, ropinirole, rotigotine, salmeterol, sotalol, tamsulosin, terazosin, terbutaline, thioridazine, timolol, trazodone, trifluoperazine, trimipramine, verapamil, ziprasidone | 1.18 (1.15, 1.21) | 1.07 (1.02, 1.12) | 0.99 (0.88, 1.11) | 1.19 (1.11, 1.26) | 1.11 |
| Insulin-like growth factor 1 receptor (other) | Insulin glulisine | 1.17 (1.12, 1.22) | 1.24 (1.15, 1.35) | 0.81 (0.64, 1.02) | 1.24 (1.11, 1.37) | 1.12 |
| Isoleucine--tRNA ligase (blocker) | Mupirocin | 1.07 (1.01, 1.13) | 1.18 (1.07, 1.31) | 1.05 (0.81, 1.36) | 1.18 (1.03, 1.35) | 1.12 |
| Calcitonin receptor (activator) | Pramlintide, salmon calcitonin | 1.21 (1.10, 1.32) | 0.95 (0.81, 1.12) | 1.39 (0.94, 2.06) | 0.97 (0.78, 1.22) | 1.13 |
| Potassium-transporting ATPase alpha chain 1 (blocker) | Esomeprazole, lansoprazole, omeprazole, pantoprazole, rabeprazole | 1.23 (1.20, 1.27) | 1.12 (1.07, 1.18) | 1.00 (0.88, 1.13) | 1.18 (1.10, 1.25) | 1.13 |
| Folate receptor (other) | Folic acid | 1.09 (0.99, 1.21) | 1.26 (1.05, 1.50) | 0.95 (0.58, 1.56) | 1.24 (0.99, 1.57) | 1.14 |
| Leukotriene A-4 hydrolase (blocker) | Captopril | 1.08 (0.96, 1.23) | 1.13 (0.92, 1.40) | 0.97 (0.53, 1.76) | 1.37 (1.06, 1.77) | 1.14 |
| DNA (other) | Altretamine, carboplatin, chlorambucil, cyclophosphamide, entecavir, gemcitabine, hydroxychloroquine, lamivudine, mechlorethamine, melphalan, methoxsalen, mitomycin, procarbazine, satraplatin, silver sulfadiazine, taribavirin, telbivudine, thalidomide, tinidazole, trifluridine, valganciclovir | 1.20 (1.11, 1.31) | 1.28 (1.11, 1.48) | 1.06 (0.72, 1.55) | 1.04 (0.85, 1.28) | 1.15 |
| Sodium-dependent noradrenaline transporter (blocker) | Amitriptyline, amoxapine, atomoxetine, benzatropine, chlorpheniramine, clomipramine, cyclobenzaprine, desipramine, dexmethylphenidate, dextroamphetamine, dextromethorphan, duloxetine, econazole, escitalopram, imipramine, maprotiline, meperidine, methylphenidate, nefazodone, nortriptyline, orphenadrine, paroxetine, protriptyline, pseudoephedrine, sertraline, tramadol, trimipramine, venlafaxine | 1.18 (1.14, 1.22) | 1.11 (1.04, 1.18) | 1.08 (0.93, 1.26) | 1.24 (1.15, 1.34) | 1.15 |
| Sodium channel (blocker) | Amiloride, benzocaine, benzonatate, bupivacaine, carbamazepine, chloroprocaine, flecainide, lamotrigine, lidocaine, mephenytoin, orphenadrine, oxcarbazepine, phenazopyridine, phenytoin, prilocaine, procainamide, promethazine, propafenone, quinidine, ranolazine, riluzole, topiramate, tramadol, triamterene, valproic acid, zonisamide | 1.23 (1.20, 1.27) | 1.14 (1.09, 1.20) | 1.05 (0.92, 1.18) | 1.20 (1.13, 1.29) | 1.16 |
| Protein S100 (calcium binding cytosolic protein family) (blocker) | Amlexanox, olopatadine, trifluoperazine, zinc chloride | 1.20 (1.12, 1.29) | 1.05 (0.92, 1.20) | 1.30 (0.96, 1.75) | 1.11 (0.93, 1.31) | 1.17 |
| Insulin-degrading enzyme (blocker) | Bacitracin | 1.18 (1.04, 1.33) | 0.91 (0.71, 1.16) | 1.23 (0.73, 2.10) | 1.35 (1.03, 1.76) | 1.17 |
| Lymphocyte antigen 96 (activator) | Morphine | 1.14 (1.01, 1.28) | 1.23 (0.99, 1.52) | 1.25 (0.77, 2.02) | 1.07 (0.80, 1.43) | 1.17 |
| Calmodulin (blocker) | Chlorpromazine, fluphenazine, nifedipine, perphenazine, phenoxybenzamine, pimozide, promethazine, trifluoperazine | 1.20 (1.14, 1.25) | 1.17 (1.08, 1.28) | 0.93 (0.74, 1.17) | 1.36 (1.22, 1.52) | 1.17 |
| Mineralocorticoid receptor (other) | Progesterone, testosterone | 1.23 (1.06, 1.43) | 0.70 (0.44, 1.12) | 1.43 (0.82, 2.48) | 1.36 (0.92, 2.00) | 1.18 |
| Retinoic acid receptor (activator) | Acitretin, adapalene, bexarotene, tazarotene, tretinoin | 1.39 (1.27, 1.53) | 1.16 (0.96, 1.40) | 0.81 (0.48, 1.37) | 1.37 (1.09, 1.73) | 1.18 |
| Neurokinin 1 receptor (blocker) | Aprepitant, tramadol | 1.20 (1.16, 1.25) | 1.14 (1.07, 1.22) | 1.16 (0.98, 1.37) | 1.23 (1.13, 1.34) | 1.18 |
| Adenosine receptor A (activator) | Gabapentin, tramadol | 1.20 (1.16, 1.25) | 1.14 (1.07, 1.22) | 1.17 (0.99, 1.38) | 1.23 (1.13, 1.35) | 1.19 |
| Transient receptor potential (TRP) channel (activator) | Acetaminophen, tramadol | 1.20 (1.16, 1.25) | 1.14 (1.07, 1.22) | 1.17 (0.99, 1.38) | 1.23 (1.13, 1.35) | 1.19 |
| Lanosterol 14-alpha demethylase (blocker) | Itraconazole, ketoconazole | 1.06 (0.96, 1.18) | 1.12 (0.92, 1.36) | 1.65 (1.13, 2.41) | 0.99 (0.76, 1.28) | 1.21 |
| Arginase-1 (other) | Urea | 1.18 (1.07, 1.29) | 1.22 (1.03, 1.44) | 1.14 (0.74, 1.74) | 1.29 (1.05, 1.60) | 1.21 |
| Steroid 21-hydroxylase (blocker) | Ketoconazole | 1.31 (1.22, 1.40) | 1.28 (1.13, 1.45) | 0.97 (0.69, 1.37) | 1.28 (1.09, 1.51) | 1.21 |
| Gamma-aminobutyric acid receptor (activator) | Acamprosate, alprazolam, arbaclofen, baclofen, butabarbital, butalbital, carisoprodol, chlordiazepoxide, clonazepam, delorazepam, diazepam, estazolam, eszopiclone, ivermectin, meprobamate, oxazepam, phenobarbital, primidone, temazepam, topiramate, triazolam, zaleplon, zolpidem | 1.27 (1.22, 1.31) | 1.22 (1.14, 1.30) | 1.08 (0.92, 1.26) | 1.26 (1.16, 1.37) | 1.21 |
| Ergosterol (other) | Butoconazole, nystatin | 1.22 (1.17, 1.28) | 1.23 (1.13, 1.33) | 1.09 (0.88, 1.36) | 1.32 (1.19, 1.48) | 1.22 |
| Fibroblast growth factor (activator) | Sucralfate | 1.28 (1.17, 1.39) | 1.10 (0.93, 1.30) | 1.26 (0.87, 1.83) | 1.25 (1.02, 1.54) | 1.22 |
| HERG human cardiac K+ channel (blocker) | Amiodarone, amitriptyline, doxazosin | 1.30 (1.23, 1.38) | 1.22 (1.10, 1.36) | 1.12 (0.86, 1.47) | 1.28 (1.11, 1.47) | 1.23 |
| Calcium channel (other) | Calcium, praziquantel, pregabalin | 1.31 (1.23, 1.39) | 1.11 (0.99, 1.25) | 1.32 (1.02, 1.71) | 1.21 (1.04, 1.40) | 1.24 |
| Gamma-aminobutyric acid receptor (other) | Alprazolam, amoxapine, carisoprodol, chlordiazepoxide, clonazepam, delorazepam, diazepam, estazolam, lindane, oxazepam, temazepam, triazolam | 1.25 (1.15, 1.36) | 1.22 (1.04, 1.44) | 1.36 (0.97, 1.91) | 1.15 (0.93, 1.43) | 1.25 |
| Ribosomal protein (other) | Citric acid, gentamicin, tigecycline | 1.21 (1.09, 1.33) | 1.36 (1.15, 1.62) | 1.22 (0.78, 1.90) | 1.21 (0.96, 1.53) | 1.25 |
| Histamine receptor (blocker) | Acrivastine, amitriptyline, amoxapine, aripiprazole, azelastine, benzatropine, brompheniramine, carbinoxamine, cetirizine, chlorpheniramine, chlorpromazine, cimetidine, clemastine, clozapine, cyproheptadine, desipramine, desloratadine, dexbrompheniramine, diphenhydramine, doxepin, econazole, epinastine, escitalopram, esmirtazapine, famotidine, fexofenadine, hydroxyzine, imipramine, ketotifen, lamotrigine, levocetirizine, loratadine, maprotiline, meclizine, mepyramine, mirtazapine, nizatidine, nortriptyline, olanzapine, olopatadine, orphenadrine, paliperidone, paroxetine, perospirone, phenyltoloxamine, prochlorperazine, promethazine, quetiapine, ranitidine, risperidone, trazodone, trimipramine, ziprasidone | 1.25 (1.21, 1.28) | 1.28 (1.22, 1.34) | 0.98 (0.87, 1.11) | 1.50 (1.41, 1.60) | 1.25 |
| Nuclear receptor (blocker) | Ketoconazole, meclizine, megestrol acetate | 1.27 (1.22, 1.33) | 1.31 (1.21, 1.41) | 0.94 (0.75, 1.18) | 1.60 (1.45, 1.76) | 1.28 |
| Adenosine deaminase (blocker) | Dipyridamole | 1.31 (1.18, 1.44) | 1.09 (0.91, 1.31) | 1.07 (0.65, 1.75) | 1.80 (1.49, 2.17) | 1.32 |
| Dopamine receptor (blocker)^b^ | Amoxapine, aripiprazole, bromocriptine, buspirone, chlorpromazine, clozapine, droperidol, escitalopram, fluphenazine, haloperidol, lamotrigine, metoclopramide, nortriptyline, olanzapine, paliperidone, perospirone, perphenazine, pimozide, prochlorperazine, promethazine, quetiapine, risperidone, thioridazine, thiothixene, trifluoperazine, ziprasidone | 1.45 (1.39, 1.51) | 1.36 (1.26, 1.46) | 0.95 (0.77, 1.17) | 1.81 (1.66, 1.99) | 1.39 |
| Sodium-dependent serotonin transporter (blocker) | Amitriptyline, amoxapine, benzatropine, chlorpheniramine, citalopram, clomipramine, cyclobenzaprine, desipramine, dexmethylphenidate, dextromethorphan, duloxetine, escitalopram, fluoxetine, fluvoxamine, imipramine, nefazodone, nortriptyline, paroxetine, protriptyline, pseudoephedrine, sertraline, tramadol, trazodone, trimipramine, venlafaxine | 1.43 (1.39, 1.48) | 1.43 (1.35, 1.50) | 1.23 (1.07, 1.41) | 1.51 (1.41, 1.62) | 1.40 |
| Acetylcholine receptor (blocker) | Amantadine, amoxapine, atropine, benzatropine, brompheniramine, bupropion, butabarbital, butalbital, chlorpromazine, clozapine, cyclopentolate, cyproheptadine, darifenacin, desipramine, dextromethorphan, dicyclomine, diphenhydramine, econazole, escitalopram, fluoxetine, glycopyrronium, homatropine, hyoscyamine, imipramine, ipratropium, lamotrigine, maprotiline, mepenzolate, methscopolamine bromide, nicardipine, nortriptyline, olanzapine, oxybutynin, paroxetine, phenobarbital, primidone, procyclidine, promethazine, propantheline, quetiapine, scopolamine, solifenacin, tiotropium, tolterodine, trihexyphenidyl, tropicamide, ziprasidone | 1.49 (1.45, 1.53) | 1.38 (1.32, 1.45) | 1.16 (1.03, 1.30) | 1.62 (1.53, 1.73) | 1.41 |
| Evolved beta-galactosidase subunit alpha (other) | Lactulose | 1.53 (1.42, 1.64) | 1.49 (1.31, 1.69) | 1.12 (0.76, 1.64) | 1.73 (1.48, 2.02) | 1.47 |
| Sigma receptor (blocker) | Sertraline | 1.40 (1.33, 1.47) | 1.76 (1.63, 1.91) | 1.18 (0.94, 1.49) | 1.64 (1.47, 1.82) | 1.50 |
| 5-hydroxytryptamine receptor (blocker) | Alosetron, amitriptyline, amoxapine, aripiprazole, bromocriptine, bupropion, cabergoline, chlorpromazine, clomipramine, clozapine, cyclobenzaprine, cyproheptadine, desipramine, dolasetron, epinastine, escitalopram, esmirtazapine, fluoxetine, granisetron, imipramine, lamotrigine, maprotiline, metoclopramide, mirtazapine, nefazodone, nortriptyline, olanzapine, ondansetron, paliperidone, palonosetron, penbutolol, perospirone, quetiapine, risperidone, thioridazine, thiothixene, tramadol, trazodone, trimipramine, ziprasidone | 1.52 (1.48, 1.57) | 1.55 (1.47, 1.63) | 1.16 (1.01, 1.33) | 1.79 (1.67, 1.91) | 1.51 |
| Histamine receptor (other) | Citalopram, amitriptyline, amoxapine, aripiprazole, chlorpromazine, haloperidol | 1.67 (1.58, 1.78) | 1.80 (1.63, 1.99) | 1.29 (0.97, 1.73) | 2.06 (1.82, 2.34) | 1.71 |
| Acetylcholine receptor (other) | Amitriptyline, aripiprazole, bethanechol, carbamazepine, choline salicylate, galantamine, glycopyrronium, haloperidol, meperidine, quetiapine, scopolamine, tiotropium, trimipramine | 1.34 (1.21, 1.49) | 2.29 (1.96, 2.67) | 0.79 (0.44, 1.43) | 2.62 (2.17, 3.16) | 1.76 |
| 5-hydroxytryptamine receptor (other) | Amitriptyline, aripiprazole, chlorpromazine, cyproheptadine, desipramine, ergotamine, fluphenazine, haloperidol, imipramine, maprotiline, methylphenidate, ondansetron, paliperidone, propranolol, quetiapine, trimipramine | 2.80 (2.64, 2.97) | 1.18 (1.02, 1.37) | 1.24 (0.87, 1.77) | 2.27 (1.95, 2.63) | 1.87 |
| Dopamine receptor (other) | Amphetamine, aripiprazole, chlorpromazine, desipramine, ergoloid mesylate, imipramine, maprotiline, paroxetine, quetiapine, trimipramine | 1.83 (1.75, 1.91) | 2.08 (1.94, 2.24) | 1.14 (0.89, 1.44) | 2.44 (2.23, 2.67) | 1.87 |
| Dopamine receptor (activator)^b^ | Amantadine, bromocriptine, cabergoline, ergotamine, pramipexole, ropinirole, rotigotine | 2.65 (2.47, 2.85) | 1.12 (0.93, 1.34) | 1.58 (1.09, 2.29) | 2.30 (1.93, 2.74) | 1.91 |
| Sodium-dependent dopamine transporter (blocker) | Amphetamine, armodafinil, benzatropine, bupropion, chloroprocaine, chlorpheniramine, dexmethylphenidate, dextroamphetamine, duloxetine, escitalopram, imipramine, meperidine, methylphenidate, modafinil, nefazodone, pseudoephedrine, sertraline, trimipramine, venlafaxine | 2.08 (1.99, 2.18) | 1.90 (1.73, 2.08) | 1.33 (1.05, 1.68) | 2.47 (2.22, 2.75) | 1.95 |
| Cholinesterase (blocker) | Dipivefrin, donepezil, echothiophate, galantamine, malathion, neostigmine, pyridostigmine, ranitidine, rivastigmine | 1.44 (1.38, 1.51) | 3.74 (3.54, 3.96) | 0.86 (0.67, 1.09) | 3.62 (3.36, 3.89) | 2.42 |
| 5-hydroxytryptamine receptor (activator) | Almotriptan, aripiprazole, bromocriptine, buspirone, cabergoline, dihydroergotamine, donepezil, eletriptan, ergotamine, frovatriptan, imipramine, metoclopramide, naratriptan, ondansetron, paroxetine, perospirone, pramipexole, rizatriptan, rotigotine, sumatriptan, trimipramine, ziprasidone, zolmitriptan | 2.03 (1.96, 2.11) | 3.46 (3.27, 3.66) | 1.16 (0.95, 1.41) | 3.87 (3.60, 4.16) | 2.63 |
| ^a^ OR comparing those who did vs. did not use any of the listed medications prior to diagnosis/reference, adjusted for age (continuous, using Harrell’s method), sex, race/ethnicity (4 categories: White, Black, Hispanic, and API/other/unknown), use of medical care (total number of physician/outpatient visits, total number of days in a hospital/skilled nursing facility, and total number of medical conditions, each prior to the case diagnosis or control reference, as a continuous variable), and a dichotomous indicator of smoking (lung cancer or chronic obstructive pulmonary disease).  ^b^ Some medications are listed as both blockers and activators for the same target. This occurs when there are multiple subtypes for the target and the effect differs by subtype, such as for adrenergic receptors and dopamine receptors.  Abbreviations: AD=Alzheimer disease; ALS=amyotrophic lateral sclerosis; API=Asian/Pacific Islander; CI=confidence interval; OR=odds ratio; PD=Parkinson disease. | | | | | | |

**S4. Table Carvedilol, other beta blockers, and risk of neurodegenerative disease, U.S. Medicare 2010-2014**

| **PD/AD/ALS in specified years of follow up** | | **Total** | **Incident PD/AD/ALS** | **Carvedilol category** | | **Any carvedilol** | |
| --- | --- | --- | --- | --- | --- | --- | --- |
|  |  |  |  | **Non-exposed comparator**  **HR (95% CI)**^a^ | **Active comparator**  **HR (95% CI)**^a^ | **Basic model**  **HR (95% CI)**^a^ | **Full model**  **HR (95% CI)**^a,b^ |
| **Years 1-5 (all years)** | | **N =** **207,764**  **n (%)** | **N =** **18,293**  **n (%)** |  |  |  |  |
|  | No beta blockers | 124,717 (60.0) | 10,430 (57.0) | 1.0 (Reference) | **--** | 1.0 (Reference) | 1.0 (Reference) |
|  | Beta blockers, no carvedilol | 74,327 (35.8) | 7,030 (38.4) | 0.99 (0.96, 1.02)^c^ | 1.0 (Reference) |  |  |
|  | Carvedilol and other beta blocker(s) | 2,606 (1.3) | 281 (1.5) | 1.06 (0.94, 1.19) | 1.07 (0.95, 1.20) | 0.98 (0.91, 1.05) | 0.97 (0.90, 1.04) |
|  | Carvedilol only | 6,114 (2.9) | 552 (3.0) | 0.93 (0.86, 1.02) | 0.94 (0.86, 1.03) |  |  |
| **Years 2-5** | | **N =** **203,470**^d^  **n (%)** | **N =** **13,999**  **n (%)** |  |  |  |  |
|  | No beta blockers | 122,351 (60.1) | 8,064 (57.6) | 1.0 (Reference) | **--** | 1.0 (Reference) | 1.0 (Reference) |
|  | Beta blockers, no carvedilol | 72,619 (35.7) | 5,322 (38.0) | 0.97 (0.94, 1.01)^c^ | 1.0 (Reference) |  |  |
|  | Carvedilol and other beta blocker(s) | 2,517 (1.2) | 192 (1.4) | 0.97 (0.84, 1.12) | 0.99 (0.86, 1.15) | 0.96 (0.89, 1.05) | 0.96 (0.89, 1.05) |
|  | Carvedilol only | 5,983 (2.9) | 421 (3.0) | 0.94 (0.86, 1.04) | 0.97 (0.88, 1.07) |  |  |
| **Years 3-5** | | **N =** **199,496**^d^  **n (%)** | **N = 10,025**  **n (%)** |  |  |  |  |
|  | No beta blockers | 120,109 (60.2) | 5,822 (58.1) | 1.0 (Reference) | **--** | 1.0 (Reference) | 1.0 (Reference) |
|  | Beta blockers, no carvedilol | 71,084 (35.6) | 3,787 (37.8) | 0.96 (0.92, 1.001)^c^ | 1.0 (Reference) |  |  |
|  | Carvedilol and other beta blocker(s) | 2,462 (1.2) | 137 (1.4) | 0.98 (0.82, 1.16)^c^ | 1.02 (0.86, 1.21)^c^ | 0.93 (0.84, 1.02) | 0.95 (0.86, 1.05) |
|  | Carvedilol only | 5,841 (2.9) | 279 (2.8) | **0.88 (0.78, 0.998)** | 0.92 (0.81, 1.04) |  |  |
| **Years 4-5** | | **N =** **195,717**^d^  **n (%)** | **N =** **6,246**  **n (%)** |  |  |  |  |
|  | No beta blockers | 117,905 (60.2) | 3,618 (57.9) | 1.0 (Reference) | **--** | 1.0 (Reference) | 1.0 (Reference) |
|  | Beta blockers, no carvedilol | 69,689 (35.6) | 2,392 (38.3) | 0.98 (0.93, 1.03) | 1.0 (Reference) |  |  |
|  | Carvedilol and other beta blocker(s) | 2,402 (1.2) | 77 (1.2) | 0.91 (0.72, 1.14) | 0.93 (0.74, 1.17) | **0.87 (0.76, 0.99)** | 0.90 (0.79, 1.03) |
|  | Carvedilol only | 5,721 (2.9) | 159 (2.5) | **0.83 (0.71, 0.98)** | 0.85 (0.72, 1.001) |  |  |
| **Year 5** | | **N =** **192,514**^d^  **n (%)** | **N = 3,043**  **n (%)** |  |  |  |  |
|  | No beta blockers | 116,039 (60.3) | 1,752 (57.6) | 1.0 (Reference) | **--** | 1.0 (Reference) | 1.0 (Reference) |
|  | Beta blockers, no carvedilol | 68,483 (35.6) | 1,186 (39.0) | 1.01 (0.94, 1.09)^c^ | 1.0 (Reference) |  |  |
|  | Carvedilol and other beta blocker(s) | 2,359 (1.2) | 34 (1.1) | 0.86 (0.61, 1.21) | 0.85 (0.60, 1.20) | **0.81 (0.67, 0.99)** | 0.86 (0.70, 1.05) |
|  | Carvedilol only | 5,633 (2.9) | 71 (2.3) | 0.79 (0.62, 1.01) | **0.78 (0.62, 0.996)** |  |  |
| ^a^ With age as the time scale to account for age, and adjusted for sex, race/ethnicity (4 categories: White, Black, Hispanic, and API/other/unknown), use of medical care (total number of physician/outpatient visits, total number of days in a hospital/skilled nursing facility, and total number of medical conditions, each prior to case diagnosis or control reference as continuous variables), and indicator of smoking (lung cancer or COPD).  ^b^ Also adjusted for other individual systemic (non-ophthalmic) beta blockers (acebutolol, atenolol, betaxolol, bisoprolol, labetalol, metoprolol, nadolol, penbutolol, pindolol, propranolol, and sotalol), acute myocardial infarction, other ischemic heart disease, and congestive heart failure.  ^c^ Failed the proportional hazards assumption.  ^d^ Excludes incident PD/AD/ALS from the prior year(s).  Abbreviations: AD=Alzheimer disease; ALS=amyotrophic lateral sclerosis; API=Asian/Pacific Islander; COPD=chronic obstructive pulmonary disease; CI=confidence interval; HR=hazard ratio; PD=Parkinson disease. | | | | | | | |
